# Photodynamic Therapy as a Potent Radiosensitizer in Head and Neck Squamous Cell Carcinoma

**DOI:** 10.3390/cancers13061193

**Published:** 2021-03-10

**Authors:** Won Jin Cho, David Kessel, Joseph Rakowski, Brian Loughery, Abdo J. Najy, Tri Pham, Seongho Kim, Yong Tae Kwon, Ikuko Kato, Harold E. Kim, Hyeong-Reh C. Kim

**Affiliations:** 1 Department of Pathology, Wayne State University School of Medicine, Detroit, MI 48201, USA; wcho@med.wayne.edu (W.J.C.); anajy@med.wayne.edu (A.J.N.); tpham@med.wayne.edu (T.P.); 2 Department of Pharmacology, Wayne State University School of Medicine, Detroit, MI 48201, USA; 3 Department of Oncology, Karmanos Cancer Institute, Wayne State University School of Medicine, Detroit, MI 48201, USA; rakowski@karmanos.org (J.R.); lougherb@karmanos.org (B.L.); kimse@karmanos.org (S.K.); katoi@karmanos.org (I.K.); kimh@karmanos.org (H.E.K.); 4 Division of Radiation Oncology, Karmanos Cancer Institute, Wayne State University School of Medicine, Detroit, MI 48201, USA; 5 Department of Biomedical Sciences, College of Medicine, Seoul National University, Seoul 03080, Korea; yok5@snu.ac.kr

**Keywords:** photodynamic therapy, ER/mitochondrial photosensitizer, paraptosis, apoptosis, autophagy

## Abstract

**Simple Summary:**

Despite the advances in multimodality treatment strategies, more than 30% of patients with advanced head and neck squamous cell carcinoma (HNSCC) experience recurrence of the disease that is usually derived from the residual tumor. The goal of our study is to understand the molecular basis underlying radiotherapy resistance in advanced HNSCC and to identify a mechanism-based radiosensitizer. We found that the autophagic cell survival pathway is upregulated in therapy-resistant HNSCC. Photodynamic therapy (PDT) directed at the endoplasmic reticulum (ER)/mitochondria induces programmed cell death such as paraptosis and apoptosis in an autophagic adaptor p62-dependent manner, promoting radiotoxicity.

**Abstract:**

Despite recent advances in therapeutic modalities such as radiochemotherapy, the long-term prognosis for patients with advanced head and neck squamous cell carcinoma (HNSCC), especially nonviral HNSCC, remains very poor, while survival of patients with human papillomavirus (HPV)-associated HNSCC is greatly improved after radiotherapy. The goal of this study is to develop a mechanism-based treatment protocol for high-risk patients with HPV-negative HNSCC. To achieve our goal, we have investigated molecular mechanisms underlying differential radiation sensitivity between HPV-positive and -negative HNSCC cells. Here, we found that autophagy is associated with radioresistance in HPV-negative HNSCC, whereas apoptosis is associated with radiation sensitive HPV-positive HNSCC. Interestingly, we found that photodynamic therapy (PDT) directed at the endoplasmic reticulum (ER)/mitochondria initially induces paraptosis followed by apoptosis. This led to a substantial increase in radiation responsiveness in HPV-negative HNSCC, while the same PDT treatment had a minimal effect on HPV-positive cells. Here, we provide evidence that the autophagic adaptor p62 mediates signal relay for the induction of apoptosis, promoting ionizing radiation (XRT)-induced cell death in HPV-negative HNSCC. This work proposes that ER/mitochondria-targeted PDT can serve as a radiosensitizer in intrinsically radioresistant HNSCC that exhibits an increased autophagic flux.

## 1. Introduction

Organ-sparing neoadjuvant chemoradiotherapy is an emerging treatment for patients with advanced head and neck squamous cell carcinoma (HNSCC). Despite the advances in multimodality treatment strategies, patients with advanced HNSCC often experience recurrence of the disease, usually derived from residual tumor [1]. HNSCC can be divided into two groups based on the status of human papillomavirus (HPV) infection [2,3,4]. Clinical characteristics of HPV-associated HNSCC are its greater sensitivity to radiotherapy [5,6,7] and better survival of patients compared to those with HPV-negative HNSCC [5,6,7,8,9,10,11,12]. To develop a mechanism-based radiosensitizer for intrinsically radioresistant HNSCC cells, we investigated the molecular basis underlying differential responses to ionizing radiation therapy (XRT) in radiosensitive HPV-positive and radioresistant HPV-negative HNSCC cell lines.

Radiation-induced tumor cell toxicity is attributable to cell death through mitotic catastrophe when cells attempt to divide with unrepaired DNA damage. However, increasing evidence suggest that tumor responses to radiotherapy are greatly affected by tumor cell sensitivity to programmed cell death (PCD) and their interactions with survival pathways [13,14]. Although apoptosis is a frequently observed PCD mode, recent studies have identified another PCD pathway termed paraptosis. It is characterized by a pattern of cytoplasmic vacuolization derived from the endoplasmic reticulum (ER) [15]. In addition, autophagy is sometimes associated with cell death, although it is usually a cytoprotective mechanism [16].

In this study, we show that increased autophagic flux is associated with radioresistance in HPV-negative HNSCC, while apoptosis is associated with radiosensitivity in HPV-positive HNSCC. In search for death stimuli that effectively kill radioresistant HNSCC cells, we assessed the effect of photodynamic therapy (PDT). PDT is a procedure involving the selective photosensitization of malignant cell types using agents that, upon excitation at a wavelength corresponding to an absorbance band, convert molecular oxygen to a series of reactive oxygen species (ROS). This has been used for the successful treatment of cancers and skin conditions [17,18,19,20]. In the context of PDT, ER photodamage leads to photodamaged ER proteins, which may mimic “misfolded protein”-mediated ER stress [21]. We found that ER photodamage effectively induces cytotoxicity in HPV-negative cells. In contrast, responses to ER photodamage in HPV-positive cells are minimal for reasons not yet understood. ER-targeted PDT activates the ER stress signaling pathways in HPV-negative HNSCC, leading to paraptosis followed by apoptosis. In contrast, the low degree of efficacy of lysosomal photodamage was comparable between HPV-positive and -negative HNSCC cells. We found that PDT using an ER/mitochondria photosensitizer promoted the efficacy of XRT in radioresistant HPV-negative HNSCC cells, but this effect was not observed with agents that targeted only the ER or lysosomes. Intrinsically radiosensitive and autophagy-deficient HPV-positive HNSCC cells were resistant to ER/mitochondria-targeted PDT. Lastly, we demonstrated that the autophagy adaptor p62 is critical for the induction of apoptosis and radiosensitization in intrinsically radioresistant HNSCC cells upon ER/mitochondria-targeted PDT, whereas p62 is nonfunctional and the same treatment fails to enhance radiosensitivity in HPV-positive HNSCC cells. We propose that ER/mitochondria-targeted PDT may promote therapy responses to XRT in patients with advanced HPV-negative HNSCC with increased autophagy flux.

## 2. Materials and Methods

### 2.1. Cell Culture

Human HNSCC cell lines UP-SCC-090 and UP-SCC-154 (University of Pittsburgh, Pittsburgh, PA, USA), UM-SCC-19 (University of Michigan, Ann Arbor, MI, USA) and WSU-HN-12 (Wayne State University, Detroit, MI, USA) were cultured as previously described [22].

### 2.2. Antibodies and Reagents

A list of antibodies and reagents and their sources used in this study is provided in Table S1. 

### 2.3. Photodynamic Therapy

Cells (5 × 10^5^ cells) in 35 mm tissue culture dishes were incubated at 37 °C with 0.5 μM BPD or 20 μM NPe6 for 1 h or with 1 μM hypericin for 16 h. The medium was replaced and the dishes irradiated with a 600-watt quartz-halogen source (Cat. No. 66296-600Q-R07, Newport Corp., Irvine, CA, USA). The bandwidth was confined by interference filters (± 10 nm, Oriel, Stratford, CT, USA) to 690 (BPD), 660 (NPe6) or 600 nm (hypericin). Drug and light doses were chosen based on prior experience based on clonogenic dose-response data. Light doses were calculated using a Scientech H310 Power & Energy meter (Scientech Inc., Boulder, CO, USA).

### 2.4. Ionizing Radiation Treatment (XRT)

Cells were irradiated with 0, 2, 4 and 6 Gy using a gantry-mounted Best Theratronics Gammabeam 500 with a dose rate of 1 Gy/min. Irradiation was carried out at room temperature under atmospheric oxygen conditions. The delivered dose was confirmed with the use of a Farmer chamber.

### 2.5. Clonogenic Cell Survival Assay

Cells were grown in 6-well plates. After overnight incubation, cells were treated as indicated. After 10 days of treatments, cells were rinsed with PBS, fixed with 70% ethanol and stained with 1% Crystal violet for 2 h at room temperature. The colonies were counted using GelCount, Oxford Optronix Ltd. (Abingdon, Oxford, UK).

### 2.6. DEVDase Activity Assay

Cells were lysed with a 0.5% NP40 lysis buffer, and 50 μg of protein lysates were incubated with 10 mmol/L Ac-DEVD-AMC substrate (Sigma) at 37 °C for 2 h. Fluorescence was detected using a SpectraMax Gemini (Molecular Probes, Carlsbad, CA, USA): 360-nm excitation and 460 nm emission.

### 2.7. Detection of Splice Variants of X-Box Binding Protein-1 (XBP1) mRNA by RT-PCR Analysis

Cells treated with 0.5 μM BPD (benzoporphyrin derivative) for 1 h were exposed to the light at 22.5 mJ/cm^2^, with 20 μM NPe6 for 1 h at 30 mJ/cm^2^, or with 1 μM hypericin for 16 h at 15 mJ/cm^2^, immediately followed by XRT at 2 Gy. One day after treatments, mRNAs were isolated using an RNeasy kit (Qiagen, Hilden, Germany) and cDNAs were synthesized with an iScriptTM cDNA Synthesis Kit (Bio-Rad, Hercules, CA, USA), followed by PCR analysis using GoTaq Flexi DNA Polymerase (Promega, Madison, WI, USA). PCR primers are listed in Table S1.

### 2.8. Establishment of p62-Knockdown WSU12 Cell Line

Scrambled shRNA sequence (shScram; catalog no. RHS4346) and three shRNA against p62 GIPZ (shp62; clone ID no. V3LHS-375194, -375195, -375197) were obtained from Open Biosystems (Huntsville, AL, USA). WSU12 cells were transfected with shScram or p62-targeting shRNA vectors using Lipofectamine 2000 (Invitrogen, Carlsbad, CA, USA) and selected with 0.25 μg/mL puromycin. The resulting pooled populations were referred to as shp62–94, shp62–95 and shp62–97, respectively.

### 2.9. Statistical Analysis

All experiments were repeated at least three times, and all data are presented as the mean ± SD. Statistical analysis was performed using Student’s unpaired two-tailed *t*-test. The *p* value of <0.01(**), <0.05(*) and n.s. were considered statistically very significant, significant and not significant between studied groups, respectively. The combination index was calculated using the method of constant ratio drug combination proposed by Chou and Talalay [23]. The 95% confidence interval (CI) for the combination index was estimated using 10,000 bootstrap samples. The combination index of less than, equal to, and greater than one represented synergism, additive effect and antagonism, respectively. Statistical analyses were performed using statistical software R version 4.0.2 (R Core Team, 2020) and RStudio version 1.4.1103 (RStudio Team, 2021).

### 2.10. Immunoblot Analysis

Densitometric analysis of immunoblots was performed using the NIH ImageJ program. Values were adjusted to the proper experimental control (time or treatment condition) and displayed under each panel in Supplementary Figures (Figures S1–S3) as a fold change. The uncropped whole blots with molecular weight markers are shown in the Supplemental Materials (Figure S6).

## 3. Results

### 3.1. HPV-Negative HNSCC Cell Lines Show Increased Basal Autophagy and Fail to Undergo Apoptosis after XRT, Whereas HPV-Positive HNSCC Cell Lines Are Prone to XRT-Induced Apoptosis

**Figure 1:**
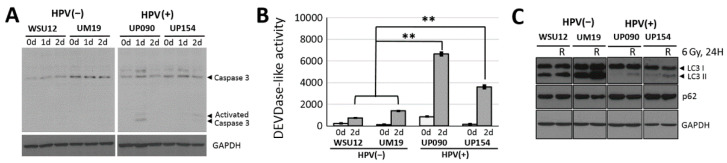
Radiosensitive human papillomavirus (HPV)-positive head and neck squamous cell carcinoma (HNSCC) cells were associated with apoptosis and radioresistant HPV-negative HNSCC with high autophagic flux. (**A**) Immunoblot of caspase 3 and (**B**) DEVDase activity assay at indicated day after irradiation at 6 Gy. (** *p* < 0.01) (**C**) Immunoblot of LC3 and p62 in control or one day after irradiation at 6 Gy.

To investigate the molecular basis for the differential radiosensitivities between HPV-positive and-negative HNSCC cells, we utilized two HPV-positive HNSCC cell lines (UP90 and UP154) and two HPV-negative HNSCC cell lines (WSU12 and UM19). HPV status was confirmed by RT-PCR analysis of the HPV oncogenes E6 and E7 in these cell lines [22]. HPV-positive UP90 and UP154 cell lines were more responsive to XRT than the HPV-negative WSU12 and UM19 cells [22]. Apoptosis, assessed by proteolytic activation of caspase 3 and DEVDase activity assay, was detected in the HPV-positive HNSCC cell lines but not in the HPV-negative HNSCC cell lines (Figure 1A,B). Differential apoptotic sensitivity was not restricted to XRT. Tumor necrosis factor-related apoptosis-inducing ligand (TRAIL) also induced caspase activity more effectively in HPV-positive HNSCC cells than in HPV-negative HNSCC cells (Figure S4). These indicated an apoptosis-resistant phenotype in HPV-negative HNSCC cell lines. In contrast to apoptotic response, autophagy was readily detected in the radioresistant HPV-negative HNSCC cells, as indicated by conversion of LC3-I to LC3-II. No evidence of autophagic flux was detected in HPV-positive cells (Figure 1C). This observation is consistent with a literature review on cytoprotective function of autophagy [24,25,26].

### 3.2. PDT Directed at ER/Mitochondria Promotes Efficacy of XRT in HPV-Negative HNSCC Cells

**Figure 2:**
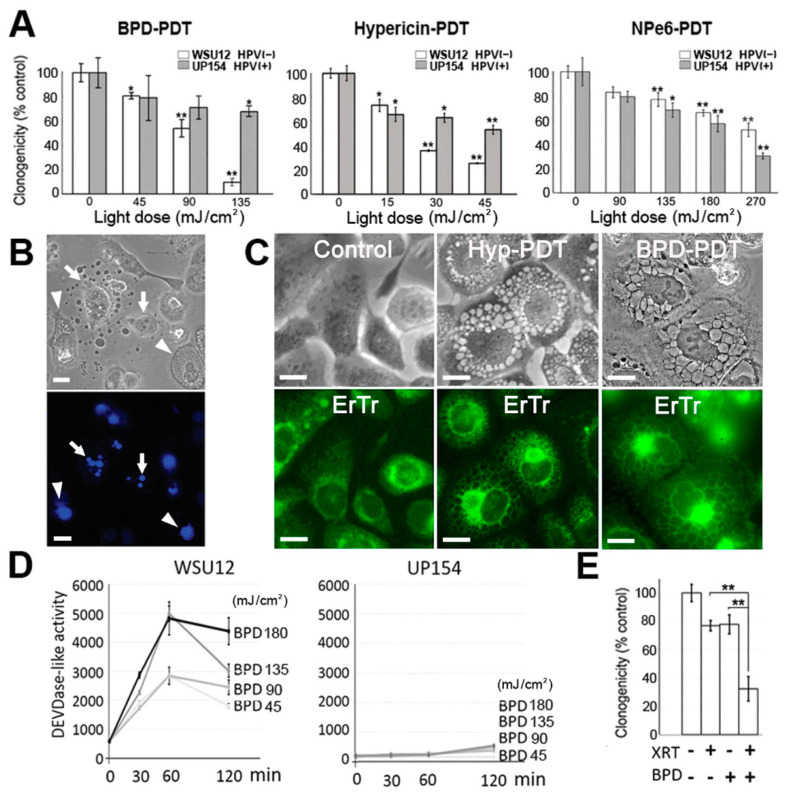
Endoplasmic reticulum (ER)/mitochondria-targeted PDT enhanced ionizing radiation therapy (XRT)-mediated cytotoxicity in HPV-negative WSU12 cells, but not in HPV-positive UP154 cells, via paraptosis and apoptosis. (**A**) Clonogenic cell survival assay. (* *p* < 0.05, ** *p* < 0.01). (**B**) Typical morphologies of apoptosis (arrow) and paraptosis (arrowhead) in WSU12 cells are shown one day after PDT. Top, phase-contrast; bottom, Hö33342 staining. Scale bar, 20 µm. (**C**) Formation of ER-derived paraptotic vacuoles in WSU12 cells after LD_90_ levels of photodamage with hypericin or BPD. Top panels, phase-contrast; bottom panels, ER Tracker. Scale bar, 20 µm. (**D**) DEVDase activity assay post BPD-PDT. (**E**) Clonogenic cell survival assay of WSU12 cells post BPD-PDT at 22.5 mJ/cm^2^, then immediately exposed to XRT at 2 Gy. (** *p* < 0.01).

Three different photosensitizing agents were examined: BPD targets both ER and mitochondria for photodamage, while hypericin is specific for the ER/Golgi and NPe6 is selective for lysosomes. Photodamage from either BPD or hypericin effectively reduced viability of HPV-negative WSU12 cells but had only a limited effect on HPV-positive UP154 cells, while NPe6 was ineffective against both cell lines (Figure 2A). Microscopic examination showed that photodamage from BPD or hypericin initially evoked paraptosis, with apoptosis observed at higher doses (Table 1). Figure 2B shows morphologic evidence for paraptosis and apoptosis in WSU12 cells after ER/mitochondrial photodamage. Paraptosis was characterized by highly-vacuolated cytoplasm but no nuclear fragmentation (Figure 2B, arrowhead). Formation of apoptotic bodies was detected by phase-contrast microscopy, and the condensed and fragmented chromatin was identified by the fluorogenic probe Hö33342 (Figure 2B bottom panel, arrow). Apoptosis in WSU12 cells was confirmed by caspase activation (Figure 2D). ER photodamage-induced paraptotic vacuoles were confirmed to be derived from ER membranes, after photodamage with hypericin or BPD, using ER Tracker Green (Figure 2C). Neither paraptosis nor apoptosis was evident in HPV-positive UP154 cells (Figure 2D and additional negative data not shown). The difference in responsiveness to photodamage from BPD was not related to differences in localized accumulation of this agent in WSU12 vs. UP154 cells (Figure S5). Photodamage from BPD was found to enhance the efficacy of XRT in WSU12 cells. An LD_20_ effect by XRT alone was increased to an LD_70_ level by addition of an LD_20_ PDT dose using BPD (Figure 2E). The combination index was 0.67 (95% Ci, 0.47 to 0.97), indicating synergism.

### 3.3. ER/mitochondria Photodamage Induces Paraptosis via JNK Activation Followed by Apoptosis Induction

**Figure 3:**
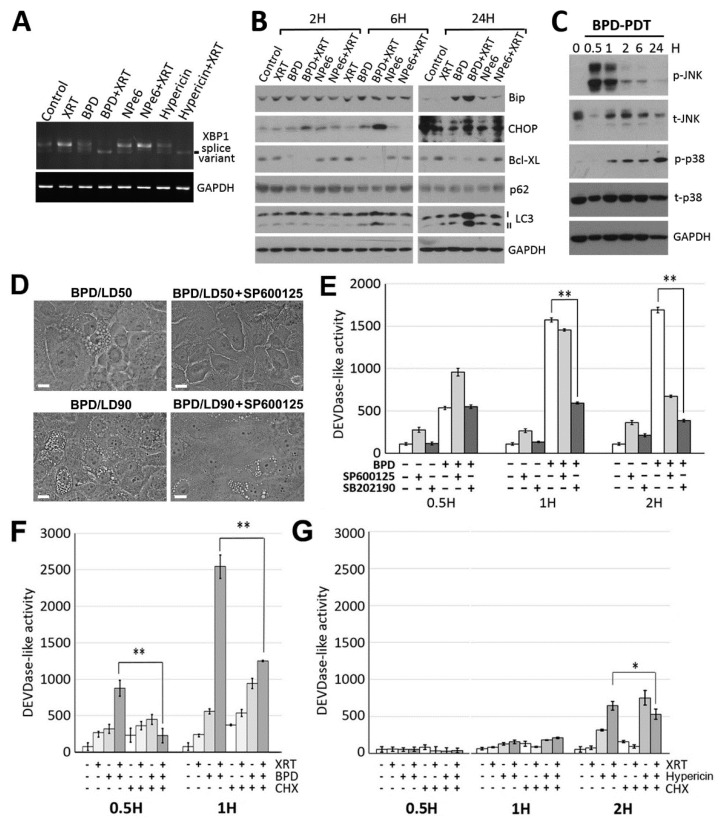
ER-targeted PDT induced ER stress signaling, and BPD-PDT sensitized WSU12 cells to XRT via apoptosis induction. (**A**) RT-PCR analysis of XBP1 splice variants in WSU12 cells upon indicated treatments. (**B**) Immunoblot of indicated proteins in WSU12 cells with BPD-PDT at 22.5 mJ/cm^2^, NPe6-PDT at 30 mJ/cm^2^ or hypericin-PDT at 15 mJ/cm^2^ with/without XRT at 2 Gy. (**C**) Immunoblot of indicated phosphorylated or total proteins in WSU12 cells with BPD-PDT at 90 mJ/cm^2^. (**D**) Phase contrast images of paraptotic WSU cells one day after BPD-PDT at LD_50_ vs. LD_90_ PDT doses with/without JNK inhibitor SP600125 (20 μM). Scale bar, 20 µm. (**E**) DEVDase activity assay in WSU12 cells after BPD-PDT at 90 mJ/cm^2^ treatment with/without indicated inhibitor. (** *p* < 0.01). (**F**,**G**) DEVDase activity assay in WSU12 cells with BPD-PDT at 22.5 mJ/cm^2^ (**F**) or Hypericin-PDT at 15 mJ/cm^2^ (**G**) with/without XRT at 2 Gy with/without cycloheximide. (* *p* < 0.05, ** *p* < 0.01).

Although little is known about the molecular pathways/mechanisms underlying paraptosis, studies have suggested that ER stress signaling pathways activate the MAPK family members that play a critical role for paraptosis [27,28,29]. ER stress signaling is mediated by three major sensors of ER stress; inositol requiring enzyme 1α (IRE1α), protein kinase RNA-activated-like ER kinase (PERK) and activating transcription factor 6α (ATF6α), whose activation is controlled by ER-resident chaperone molecule GRP78/BiP [30]. Here, we confirmed activation of ER stress signaling by BPD-PDT or hypericin-PDT as evidenced by the presence of splice variants of X-box binding protein-1 (XBP1) mRNA (Figure 3A), known to result from activation of the IRE1α pathway [30,31], as well as by upregulation of BiP and the transcription factors C/EBP homologous protein (CHOP) (Figure 3B), a downstream mediator of the PERK pathway [30,32]. Consistently with previous reports, ER stress signaling led to activation of JNKs (c-Jun N-terminal Kinases) followed by p38 activation (Figure 3C). Next, we tested the involvement of JNKs and p38 in paraptosis and apoptosis induction. Inhibition of JNKs, but not p38, prevented paraptotic cell death (Figure 3D), while inhibition of p38 (and JNK at a later time point only) reduced caspase activation (Figure 3E).

### 3.4. BPD-PDT Sensitizes WSU12 Cells to XRT via Apoptosis Induction; Implication of the Involvement of ER Stress/Paraptotic Signaling Pathways

Next, we asked whether BPD-PDT enhancement of XRT-mediated cytotoxicity is associated with apoptosis and if new protein synthesis, thought to be critical for ER stress signaling and paraptosis, is involved in apoptosis induction. To this end, we measured caspase activity after BPD-catalyzed photodamage with or without XRT in the presence or absence of cycloheximide treatment. A sublethal dose of PDT was used for better detection of its synergistic effect with XRT. While XRT alone failed to induce caspase activation in HPV-negative WSU12 cells (Figure 3F,G, second bar, and Figure 1B), XRT together with BPD-PDT effectively activated caspase (fourth bar vs. second (XRT alone) or third (BPD-PDT alone) bar in Figure 3F). Interestingly, while new protein synthesis did not seem to be required for BPD-PDT-induced caspase activation (third vs. seventh bar), cycloheximide treatment significantly prevented synergistic activation of caspase induced by cotreatment of BPD-PDT and XRT (fourth vs. eighth bar). As we previously reported [33], apoptosis induced by PDT is associated with destruction of anti-bcl-2 family member Bcl-xL (Figure 3B). We surmise that while caspase activation is independent of protein synthesis and associated with the loss of antiapoptotic Bcl-xL protein, synergistic apoptosis induction by BPD-PDT and XRT requires newly synthesized protein-mediated signaling. Hypericin-PDT was ineffective in synergistically activating caspase together with XRT (Figure 3G), consistent with clonogenic cell survival assay (Figure 2E).

### 3.5. A Critical Role of the Autophagic Adaptor p62 in the Signal Relay for BPD-PDT-Mediated Apoptosis and Radiosensitization in Nonviral HNSCC Cells

**Figure 4:**
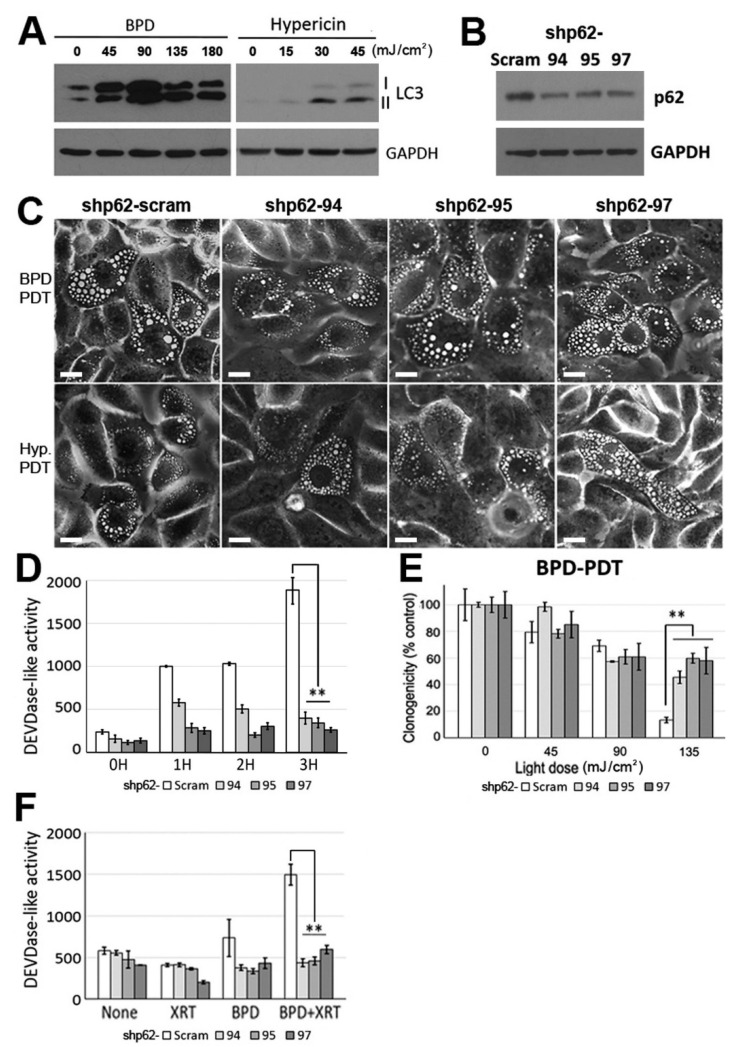
The autophagic adaptor p62 plays an important role in BPD-PDT-mediated apoptosis and radiosensitization. (**A**) Immunoblot analysis of LC3 in WSU12 cells at 1-day after post BPD-PDT or hypericin-PDT treatments with indicated light doses. (**B**) Immunoblot analysis of p62 in control (Scram) and p62 knockdown WSU12 cell lines (shp62–94, shp62–95 and shp62–97). (**C**) Phase contrast images of paraptotic cells in control and p62 knockdown WSU12 cells at one day after BPD-PDT at 90 mJ/cm^2^ or hypericin-PDT at 45 mJ/cm^2^. Scale bar, 20 µm. (**D**) DEVDase activity in control and p62 knockdown WSU12 cells upon BPD-PDT at 90 mJ/cm^2^. (** *p* < 0.01). (**E**) Clonogenic cell survival of control and p62 knockdown WSU12 cells upon BPD-PDT. (** *p* < 0.01). (**F**) DEVDase activity was measured at 4 h after treatments with/without XRT at 2 Gy and/or BPD-PDT at 180 mJ/cm^2^. (** *p* < 0.01).

Next, we asked whether differential interactions among cell death pathways attributable to differential regulation of ER/mitochondria photodamage-induced apoptosis and radiosensitization between HPV-positive and -negative HNSCC. Here we hypothesized that while high autophagic flux may contribute to apoptosis-resistant and radioresistant phenotypes in nonviral HNSCC cells (Figure 1), autophagy regulator(s) interact(s) with ER/mitochondria photodamage-induced stress signals, resulting in conversion of cell survival to cell death. First, we confirmed that ER photodamage further promotes the autophagic flux in HPV-negative WSU 12 cells, as detected by LC3-I conversion to LC3-II (Figure 4A), whereas HPV-positive cells were defective in inducing autophagy (Figure 1C). Since evidence supports that the autophagic adaptor p62 mediates ER stress signaling via its interaction with arginylated BiP (R-BiP) [34], we examined the involvement of the autophagic adaptor p62 in the regulation of paraptosis, apoptosis and cell fate upon ER/mitochondria photodamage. To this end, p62-knockdown WSU12 cell lines were established using three different vector-based short hairpin RNA (shRNA) constructs. Immunoblot analysis confirmed downregulation of p62 expression in cells receiving specific p62 target sequences (Figure 4B). While p62 knockdown had little effect on induction of paraptosis, this significantly impaired caspase activation (Figure 4C,D). Consistently, while p62 knockdown had little effect on low-dose BPD-PDT-induced cell toxicity (mainly paraptotic cell death), p62 knockdown significantly protected WSU12 cells from high-dose BPD PDT-induced cell apoptotic cell death (Figure 4E and Table 1). These results suggest that p62 functions as a signaling hub for the induction of apoptosis after photodamage. Further investigation of the involvement of p62 in apoptosis showed that p62 knockdown prevented synergistic caspase activation after ER/mitochondrial photodamage together with XRT (Figure 4F), providing molecular insight into p62-mediated radiosensitization upon ER/mitochondria photodamage in radioresistant HNSCC cells with high autophagy flux.

## 4. Discussion

**Figure 5:**
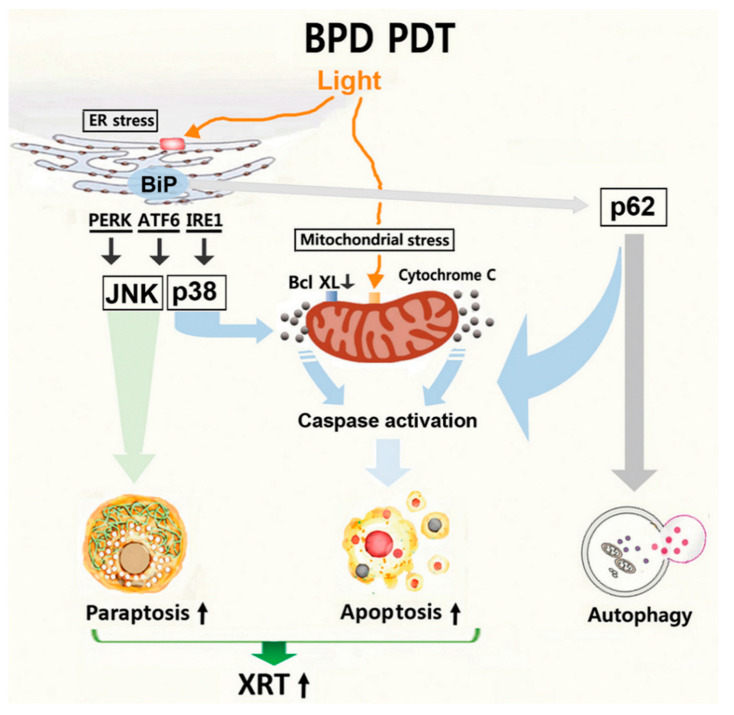
A working model of BPD-PDT-mediated paraptosis, apoptosis and sensitization to XRT.

This report identifies photodamage to ER and mitochondria using BPD, an FDA-approved photosensitizer, as a means to sensitize nonviral HNSCC cells to XRT, consistent with our previous report [35]. Our results indicate that the molecular mechanism underlying BPD-PDT-induced drastic conversion of an apoptosis-resistant phenotype to an apoptotic-responsive phenotype after XRT involves the autophagy adaptor p62. As depicted in Figure 5, ER stress signaling leads to activation of JNKs and paraptosis as well as activation of p62 and autophagy. At present, it is unclear whether ER stress-induced autophagy initiated by PDT is associated with cytoprotection [36] or with cell death. After ER photodamage, ER stress signaling and autophagic regulators may interact with the mitochondrial apoptotic pathway, resulting in activation of caspase. Similarly to our study, recent findings report signal interplay between autophagy and apoptosis [37]. Dephosphorylation and cleavage of Beclin-1 disrupt R-BiP/Beclin-1/p62 complex, thereby converting autophagy to apoptosis [37]. In this study, both apoptosis and the autophagic flux are further enhanced upon photodamage and XRT (Figure 3B and Figure 4A). These results suggest that caspase activation mediated by p62 may not occur at the expense of reducing autophagy through conversion from autophagy to apoptosis. Instead, we hypothesize that p62 functions as a signaling adaptor for apoptosis. Consistent with our hypothesis, a direct involvement of p62 in caspase activation has been suggested: the ER stress inducer tunicamycin was shown to induce apoptosis involving p62-mediated caspase-8 activation [38] and the ubiquitin-binding function of p62 promotes aggregation and activation of caspase-8 [39].

We showed that ER stress-induced paraptosis preceded apoptosis in cells where apoptosis was impaired after radiotherapy. At present, it is unclear whether PDT-induced paraptosis is a prerequisite for apoptosis induction or these are two different cell death pathways independently induced by ER/mitochondria targeted PDT. Although recent studies have reported the significance of paraptosis, for instance, as an important cell death mode upon Zika virus infection [15], its presence in vivo and the functional significance in cancer therapy remain to be investigated. In light of reports demonstrating that high tumor immunogenicity is associated with nonapoptotic death of tumor cells in vivo [40], PDT-induced paraptosis warrants further investigation.

PDT is a local treatment method, known to be selective for malignant tissues and sparing normal tissues at tumor margins [16,17,18]. Improvement of locoregional control of HNSCC is critical for the management of patients with advanced HNSCC, and the treatment of those patients with PDT has resulted in promising outcomes [41,42,43,44]. Based on our results, we propose that ER/mitochondria-targeted PDT may serve as a part of organ-sparing multimodality treatment strategies to improve the efficacy of chemoradiotherapy for patients with advanced nonviral HNSCC with high autophagic flux.

## 5. Conclusions

Autophagy is associated with radioresistance in HPV-negative HNSCC, whereas apoptosis is associated with radiation sensitive HPV-positive HNSCC. The present study identified a tool to target the survival pathway in therapy-resistant HPV-negative HNSCC. Photodynamic therapy (PDT) directed at the ER/mitochondria initially induced paraptosis followed by apoptosis. This led to a substantial increase in radiation responsiveness in HPV-negative HNSCC in an autophagic adaptor p62-dependent manner. In contrast, the same PDT treatment had a minimal effect on HPV-positive cells. This work proposes that ER/mitochondria targeted PDT can serve as a radiosensitizer in intrinsically radio-resistant HNSCC that exhibits an increased autophagic flux.

## Figures and Tables

**Table 1:** BPD-photodynamic therapy (PDT)- and hypericin-PDT-induced cell death modes.

Photosensitizing Agent	Light Dose (mJ/cm^2^)	WSU12		UP154	
		Paraptosis	Apoptosis	Paraptosis	Apoptosis
BPD	45	+	−	−	−
	90	+++	+	−	−
	135	++	+	−	−
	180	+	++	−	−
Hypericin	15	+	-	−	−
	30	+++	-	−	−
	45	++	+	−	−
	60	+	+	−	−

Percent (%) cells in each category: − (<5), + (5–10), ++ (10–30), +++ (>30).

## Data Availability

The data generated within the current study are available from the corresponding authors upon request.

## Supplementary Materials

The following are available online at https://www.mdpi.com/2072-6694/13/6/1193/s1. Figure S1: Densitometric analysis of Figure 1A,C, Figure S2: Densitometric analysis of Figure 3B,C, Figure S3: Densitometric analysis of Figure 4A,B, Figure S4: Tumor necrosis factor-related apoptosis-inducing ligand (TRAIL) induces apoptosis more effectively in HPV-positive HNSCC cells compared to HPV-negative HNSCC cells, Figure S5: Uptake and localization of BPD in WSU12 and UP154 cells, Figure S6: Uncropped entire immunoblots with molecular weight markers, Table S1: List of PCR primers, Table S2: List of antibodies and reagents.

## References

[1] Braakhuis B.J., Brakenhoff R.H., Leemans C.R. (2012). Treatment choice for locally advanced head and neck cancers on the basis of risk factors: Biological risk factors. Ann. Oncol..

[2] Attner P., Du J., Nasman A., Hammarstedt L., Ramqvist T., Lindholm J., Marklund L., Dalianis T., Munck-Wikland E. (2010). The role of human papillomavirus in the increased incidence of base of tongue cancer. Int. J. Cancer.

[3] Chaturvedi A.K., Engels E.A., Anderson W.F., Gillison M.L. (2008). Incidence trends for human papillomavirus-related and -unrelated oral squamous cell carcinomas in the United States. J. Clin. Oncol..

[4] Hammarstedt L., Lindquist D., Dahlstrand H., Romanitan M., Dahlgren L.O., Joneberg J., Creson N., Lindholm J., Ye W., Dalianis T. (2006). Human papillomavirus as a risk factor for the increase in incidence of tonsillar cancer. Int. J. Cancer.

[5] Fakhry C., Westra W.H., Li S., Cmelak A., Ridge J.A., Pinto H., Forastiere A., Gillison M.L. (2008). Improved survival of patients with human papillomavirus-positive head and neck squamous cell carcinoma in a prospective clinical trial. J. Natl. Cancer Inst..

[6] Hong A.M., Dobbins T.A., Lee C.S., Jones D., Harnett G.B., Armstrong B.K., Clark J.R., Milross C.G., Kim J., O’Brien C.J. (2010). Human papillomavirus predicts outcome in oropharyngeal cancer in patients treated primarily with surgery or radiation therapy. Br. J. Cancer.

[7] Sethi S., Ali-Fehmi R., Franceschi S., Struijk L., van Doorn L.J., Quint W., Albashiti B., Ibrahim M., Kato I. (2012). Characteristics and survival of head and neck cancer by HPV status: A cancer registry-based study. Int. J. Cancer.

[8] Ang M.K., Patel M.R., Yin X.Y., Sundaram S., Fritchie K., Zhao N., Liu Y., Freemerman A.J., Wilkerson M.D., Walter V. (2011). High XRCC1 protein expression is associated with poorer survival in patients with head and neck squamous cell carcinoma. Clin. Cancer Res..

[9] Lill C., Kornek G., Bachtiary B., Selzer E., Schopper C., Mittlboeck M., Burian M., Wrba F., Thurnher D. (2011). Survival of patients with HPV-positive oropharyngeal cancer after radiochemotherapy is significantly enhanced. Wien. Klin. Wochenschr..

[10] Mellin H., Friesland S., Lewensohn R., Dalianis T., Munck-Wikland E. (2000). Human papillomavirus (HPV) DNA in tonsillar cancer: Clinical correlates, risk of relapse, and survival. Int. J. Cancer.

[11] Sedaghat A.R., Zhang Z., Begum S., Palermo R., Best S., Ulmer K.M., Levine M., Zinreich E., Messing B.P., Gold D. (2009). Prognostic significance of human papillomavirus in oropharyngeal squamous cell carcinomas. Laryngoscope.

[12] Worden F.P., Kumar B., Lee J.S., Wolf G.T., Cordell K.G., Taylor J.M., Urba S.G., Eisbruch A., Teknos T.N., Chepeha D.B. (2008). Chemoselection as a strategy for organ preservation in advanced oropharynx cancer: Response and survival positively associated with HPV16 copy number. J. Clin. Oncol..

[13] Ow T.J., Pitts C.E., Kabarriti R., Garg M.K. (2015). Effective Biomarkers and Radiation Treatment in Head and Neck Cancer. Arch. Pathol. Lab. Med..

[14] Seshacharyulu P., Baine M.J., Souchek J.J., Menning M., Kaur S., Yan Y., Ouellette M.M., Jain M., Lin C., Batra S.K. (2017). Biological determinants of radioresistance and their remediation in pancreatic cancer. Biochim Biophys Acta. Rev. Cancer.

[15] Monel B., Compton A.A., Bruel T., Amraoui S., Burlaud-Gaillard J., Roy N., Guivel-Benhassine F., Porrot F., Genin P., Meertens L. (2017). Zika virus induces massive cytoplasmic vacuolization and paraptosis-like death in infected cells. EMBO. J..

[16] Codogno P., Meijer A.J. (2005). Autophagy and signaling: Their role in cell survival and cell death. Cell Death. Differ..

[17] Agostinis P., Berg K., Cengel K.A., Foster T.H., Girotti A.W., Gollnick S.O., Hahn S.M., Hamblin M.R., Juzeniene A., Kessel D. (2011). Photodynamic therapy of cancer: An update. CA. Cancer J. Clin..

[18] Dougherty T.J., Gomer C.J., Henderson B.W., Jori G., Kessel D., Korbelik M., Moan J., Peng Q. (1998). Photodynamic therapy. J. Natl. Cancer Inst..

[19] Kessel D., Oleinick N.L. (2010). Photodynamic therapy and cell death pathways. Methods. Mol. Biol.

[20] Del Duca E., Manfredini M., Petrini N., Farnetani F., Chester J., Bennardo L., Schipani G., Tamburi F., Sannino M., Cannarozzo G. (2019). Daylight Photodynamic Therapy with 5-aminolevulinic acid 5% gel for the treatment of mild-to-moderate inflammatory acne. G. Ital. Dermatol. Venereol..

[21] Nam J.S., Kang M.G., Kang J., Park S.Y., Lee S.J., Kim H.T., Seo J.K., Kwon O.H., Lim M.H., Rhee H.W. (2016). Endoplasmic Reticulum-Localized Iridium(III) Complexes as Efficient Photodynamic Therapy Agents via Protein Modifications. J. Am. Chem. Soc..

[22] Jung Y.S., Najy A.J., Huang W., Sethi S., Snyder M., Sakr W., Dyson G., Huttemann M., Lee I., Ali-Fehmi R. (2017). HPV-associated differential regulation of tumor metabolism in oropharyngeal head and neck cancer. Oncotarget.

[23] Chou T.C. (2006). Theoretical basis, experimental design, and computerized simulation of synergism and antagonism in drug combination studies. Pharmacol. Rev..

[24] Yang Z.J., Chee C.E., Huang S., Sinicrope F. (2011). Autophagy modulation for cancer therapy. Cancer Biol. Ther..

[25] Carew J.S., Kelly K.R., Nawrocki S.T. (2012). Autophagy as a target for cancer therapy: New developments. Cancer Manag. Res..

[26] Sui X., Chen R., Wang Z., Huang Z., Kong N., Zhang M., Han W., Lou F., Yang J., Zhang Q. (2013). Autophagy and chemotherapy resistance: A promising therapeutic target for cancer treatment. Cell Death. Dis..

[27] Sperandio S., Poksay K., de Belle I., Lafuente M.J., Liu B., Nasir J., Bredesen D.E. (2004). Paraptosis: Mediation by MAP kinases and inhibition by AIP-1/Alix. Cell Death. Differ..

[28] Xue Q., Wang X., Wang P., Zhang K., Liu Q. (2015). Role of p38MAPK in apoptosis and autophagy responses to photodynamic therapy with Chlorin e6. Photodiagnosis. Photodyn. Ther..

[29] Chen R., Duan C.Y., Chen S.K., Zhang C.Y., He T., Li H., Liu Y.P., Dai R.Y. (2013). The suppressive role of p38 MAPK in cellular vacuole formation. J. Cell Biochem..

[30] Rutkowski D.T., Kaufman R.J. (2004). A trip to the ER: Coping with stress. Trends. Cell Biol..

[31] Maurel M., Chevet E., Tavernier J., Gerlo S. (2014). Getting RIDD of RNA: IRE1 in cell fate regulation. Trends. Biochem. Sci..

[32] Rozpedek W., Pytel D., Mucha B., Leszczynska H., Diehl J.A., Majsterek I. (2016). The Role of the PERK/eIF2alpha/ATF4/CHOP Signaling Pathway in Tumor Progression During Endoplasmic Reticulum Stress. Curr. Mol. Med..

[33] Kim H.R., Luo Y., Li G., Kessel D. (1999). Enhanced apoptotic response to photodynamic therapy after bcl-2 transfection. Cancer Res..

[34] Cha-Molstad H., Sung K.S., Hwang J., Kim K.A., Yu J.E., Yoo Y.D., Jang J.M., Han D.H., Molstad M., Kim J.G. (2015). Amino-terminal arginylation targets endoplasmic reticulum chaperone BiP for autophagy through p62 binding. Nat. Cell Biol..

[35] Kessel D., Cho W.J., Rakowski J., Kim H.E., Kim H.C. (2020). Effects of HPV Status on Responsiveness to Ionizing Radiation vs Photodynamic Therapy in Head and Neck Cancer Cell lines. Photochem. Photobiol..

[36] Ogata M., Hino S., Saito A., Morikawa K., Kondo S., Kanemoto S., Murakami T., Taniguchi M., Tanii I., Yoshinaga K. (2006). Autophagy is activated for cell survival after endoplasmic reticulum stress. Mol. Cell Biol..

[37] Song X., Lee D.H., Dilly A.K., Lee Y.S., Choudry H.A., Kwon Y.T., Bartlett D.L., Lee Y.J. (2018). Crosstalk Between Apoptosis and Autophagy Is Regulated by the Arginylated BiP/Beclin-1/p62 Complex. Mol. Cancer Res..

[38] Ullman E., Pan J.A., Zong W.X. (2011). Squamous cell carcinoma antigen 1 promotes caspase-8-mediated apoptosis in response to endoplasmic reticulum stress while inhibiting necrosis induced by lysosomal injury. Mol. Cell Biol..

[39] Jin Z., Li Y., Pitti R., Lawrence D., Pham V.C., Lill J.R., Ashkenazi A. (2009). Cullin3-based polyubiquitination and p62-dependent aggregation of caspase-8 mediate extrinsic apoptosis signaling. Cell.

[40] Melcher A., Todryk S., Hardwick N., Ford M., Jacobson M., Vile R.G. (1998). Tumor immunogenicity is determined by the mechanism of cell death via induction of heat shock protein expression. Nat. Med..

[41] Biel M.A. (2010). Photodynamic therapy of head and neck cancers. Methods. Mol. Biol..

[42] Rigual N.R., Thankappan K., Cooper M., Sullivan M.A., Dougherty T., Popat S.R., Loree T.R., Biel M.A., Henderson B. (2009). Photodynamic therapy for head and neck dysplasia and cancer. Arch. Otolaryngol. Head Neck Surg..

[43] Mimikos C., Shafirstein G., Arshad H. (2016). Current state and future of photodynamic therapy for the treatment of head and neck squamous cell carcinoma. World J. Otorhinolaryngol. Head Neck Surg..

[44] van Doeveren T.E.M., Karakullukcu M.B., van Veen R.L.P., Lopez-Yurda M., Schreuder W.H., Tan I.B. (2018). Adjuvant photodynamic therapy in head and neck cancer after tumor-positive resection margins. Laryngoscope.

