# Supplementary material for: Photodynamic Therapy as a Potent Radiosensitizer in Head and Neck Squamous Cell Carcinoma
Source: Cancers (Basel). 2021 Mar 10;13(6):1193. doi: 10.3390/cancers13061193 (PMC7998908; doi:10.3390/cancers13061193)
Supplement: Supplementary file 1 [file cancers-13-01193-s001.zip › cancers-1125978 Supplementary materials-XML.docx]

Supplementary Materials

Photodynamic Therapy as a Potent Radiosensitizer in Head and Neck Squamous Cell Carcinoma

**Won Jin Cho, David Kessel, Joseph Rakowski, Brian Loughery, Abdo J. Najy, Tri Pham, Seongho Kim, Yong Tae Kwon, Ikuko Kato, Harold E. Kim, and Hyeong-Reh C. Kim**

**Table S1.** List of PCR primers.

| **Gene** | **Accession Number** | **Forward Primer (5’-3’)** | **Reverse Sequences (5’-3’)** |
| --- | --- | --- | --- |
| X-box binding protein-1 (XBP1) | NM_005080 | ACAGCGCTTGGGGATGGATG | TGACTGGGTCCAAGTTGTCC |
| GAPDH | NM_002046 | ATCACCATCTTCCAGGAGCGA | GCCAGTGAGCTTCCCGTTCA |

**Table S2.** List of Antibodies and Reagents.

| **Name** | **Company** | **Catalog Number** |
| --- | --- | --- |
| Antibodies | | |
| rabbit anti-Caspase3 | Cell Signaling Technology | #9665 |
| rabbit anti-LC3B | Cell Signaling Technology | #2775 |
| rabbit anti-BiP | Cell Signaling Technology | #3177 |
| mouse anti-CHOP | Cell Signaling Technology | #2895 |
| rabbit anti-Bcl-xL | Cell Signaling Technology | #2764 |
| rabbit anti-pJNKs | Cell Signaling Technology | #4668 |
| rabbit anti-JNKs | Cell Signaling Technology | #9258 |
| rabbit anti-p-p38 | Cell Signaling Technology | #4511 |
| rabbit anti-p38 | Cell Signaling Technology | #9212 |
| Mouse anti-p62 | AbCam | ab56416 |
| anti-GAPDH | Santa Cruz biotechnology | sc32233 |
| Reagent | | |
| JNK inhibitor SP600125 | Cayman chemical | #10010466 |
| p38 inhibitor SB202190 | Cayman chemical | #10010399 |
| ER tracker | ThermoFisher Sci. | E34251 |
| cycloheximide (CHX) | Sigma-Aldrich | #01810 |
| benzoporphyrin derivative monoacid ring A (BPD) | VWR | #76270 |
| hypericin | Beantown chemical | #225145 |
| N-aspartyl chlorin e6 (NPe6) | From Dr. Kevin Smith at Louisiana State University | |


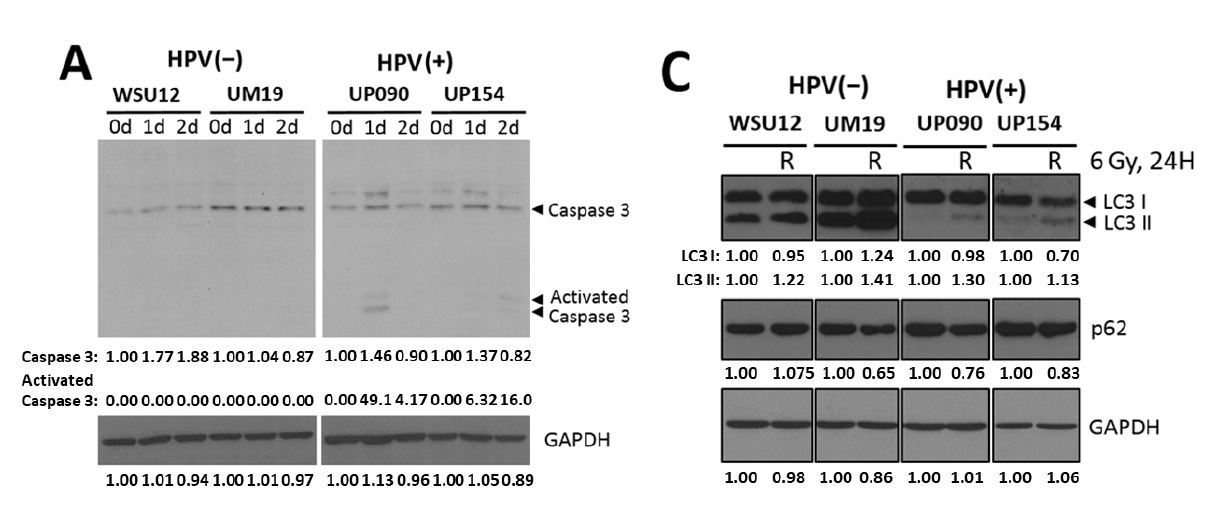


**Figure S1.** Densitometric Analysis of Figure 1A and C. Immunoblots densitometry was performed using the NIH ImageJ program. Values were adjusted to the proper experimental control (time or treatment condition) then values displayed under each panel as a fold change.


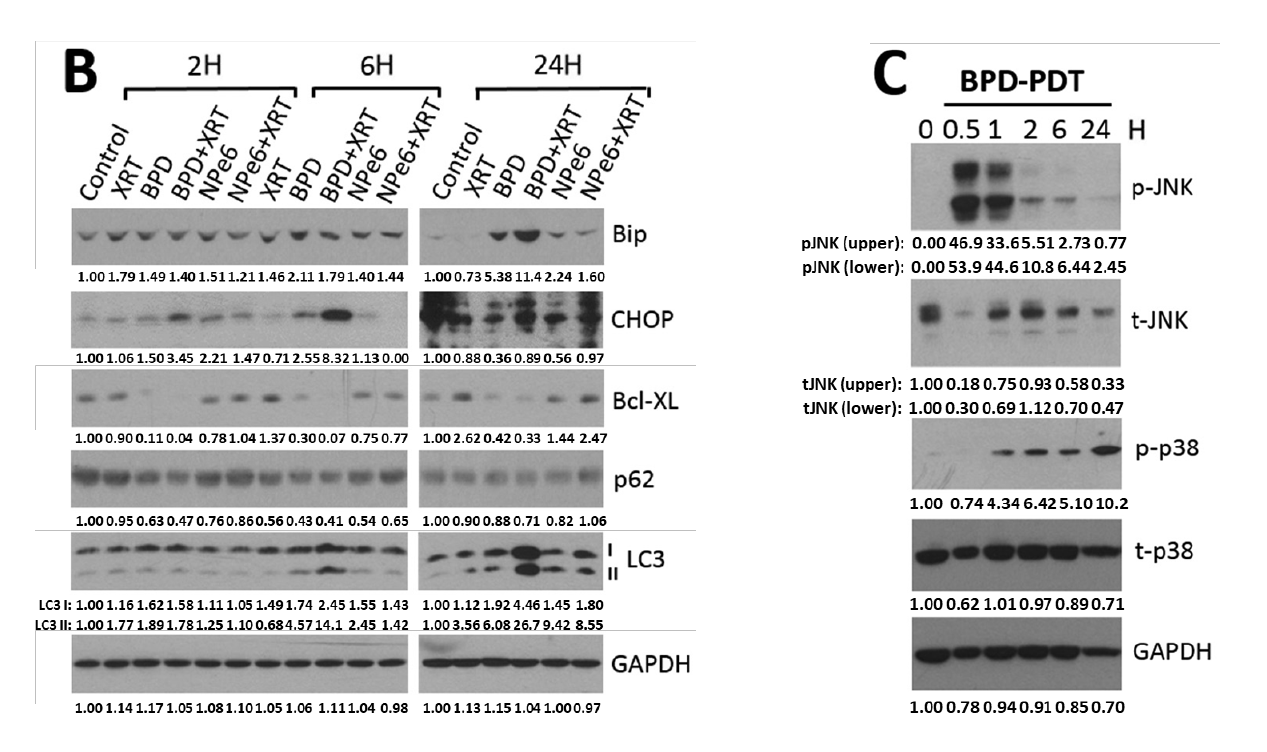


**Figure S2.** Densitometric Analysis of Figure 3B and C. Immunoblots densitometry was performed using the NIH ImageJ program. Values were adjusted to the proper experimental control (time or treatment condition) then values displayed under each panel as a fold change.


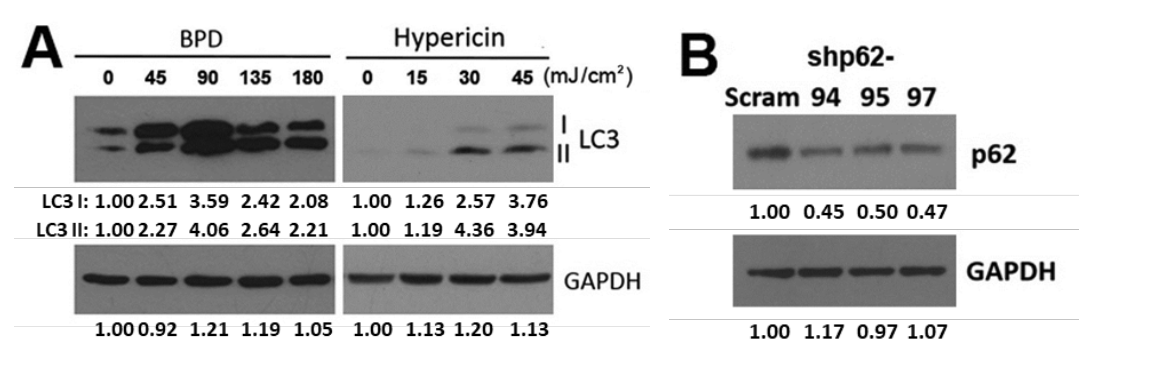


**Figure S3.** Densitometric Analysis of Figure 4A and B**.** Immunoblots densitometry was performed using the NIH ImageJ program. Values were adjusted to the proper experimental control (time or treatment condition) then values displayed under each panel as a fold change.


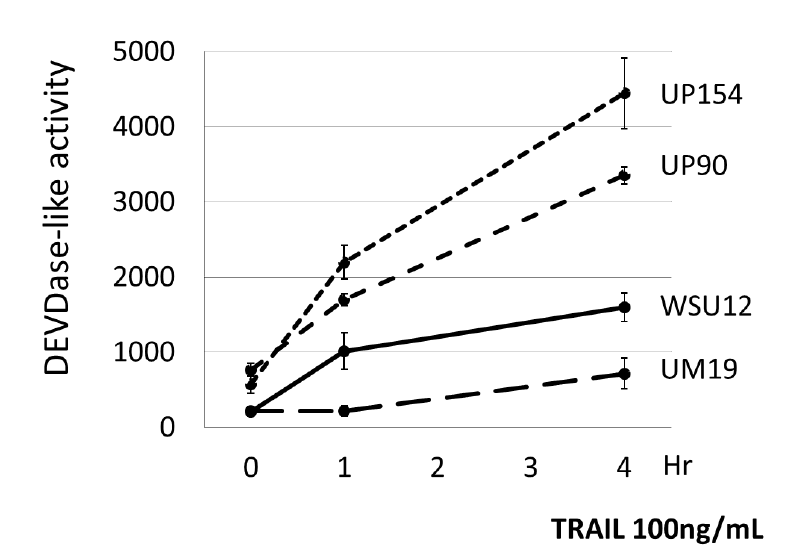


**Figure S4.** Tumor necrosis factor-related apoptosis-inducing ligand (TRAIL) induces apoptosis more effectively in HPV-positive HNSCC cells compared to HPV-negative HNSCC cells. DEVDase (Caspase 3/7) activity assay in HPV-negative cells (WSU12 and UM19) and HPV-positive cells (UP90 and UP154) at 30 min, 1 h or 4 h after TRAIL (100 ng/mL) treatment. (WSU12 *vs*. UP90, *p* < 0.05; WSU12 vs. UP154, *p* < 0.05; UM19 vs. UP90, *p* < 0.05; UM19 vs. UP154, *p* < 0.05).

**
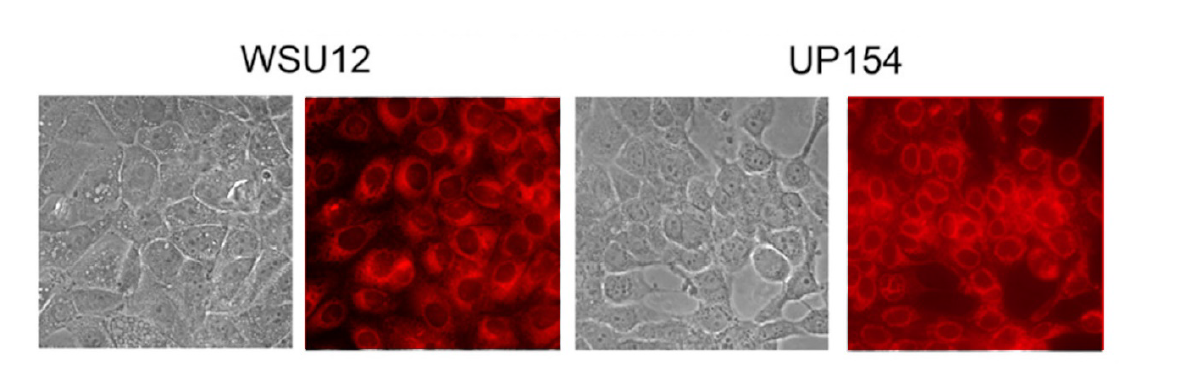
Figure S5.** Uptake and localization of BPD in WSU12 and UP154 cells. Cells were incubated with 0.5 μM BPD for 1 h at 37 °C to assess sites and relative levels of drug accumulation. Fluorescence microscopy involved excitation at 400–450 nm with fluorescence detected at 700 nm (right panels). For each cell line, left panels show phase-contrast images.

**Figure S6.**Uncropped entire immunoblots with molecular weight markers.
