# Supplementary material for: Photodynamic Therapy as a Potent Radiosensitizer in Head and Neck Squamous Cell Carcinoma
Source: Cancers (Basel). 2021 Mar 10;13(6):1193. doi: 10.3390/cancers13061193 (PMC7998908; doi:10.3390/cancers13061193)

Supplementary Figure 6

Figure 1A

Caspase3

Caspase3

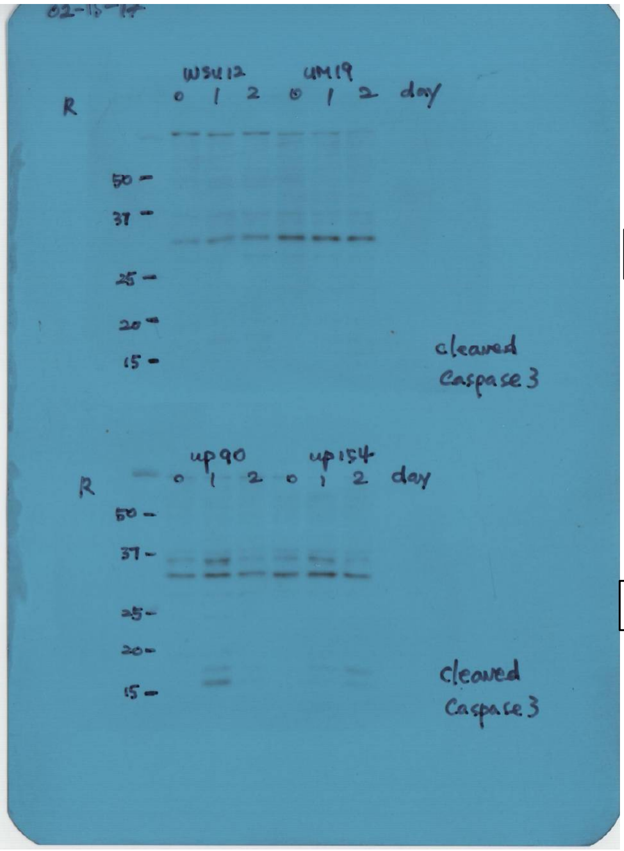

HPV(-)

Caspase3

HPV(+)

Caspase3

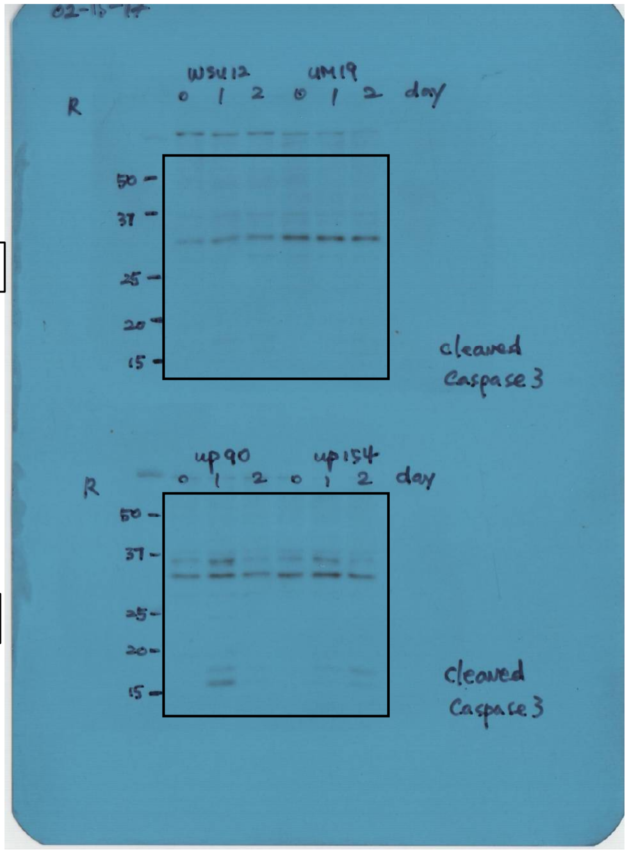

Figure 1A

GAPDH

GAPDH

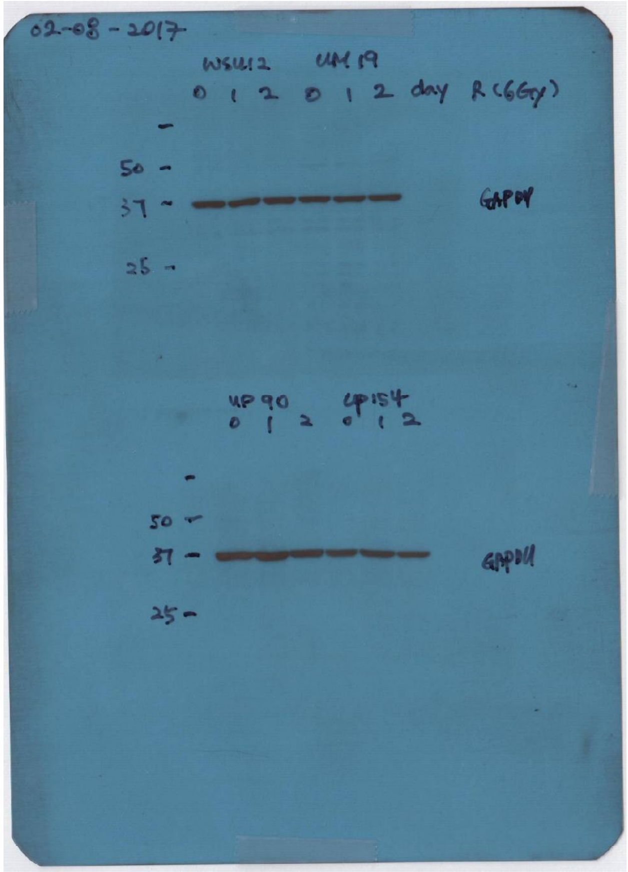

HPV(-)

GAPDH

HPV(+)

GAPDH

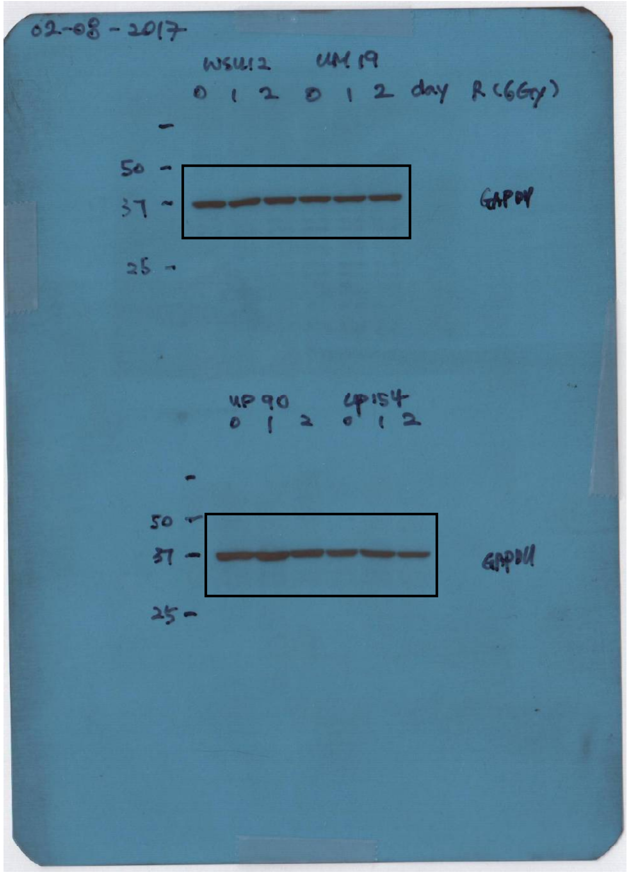

Figure 1C

LC3

LC3

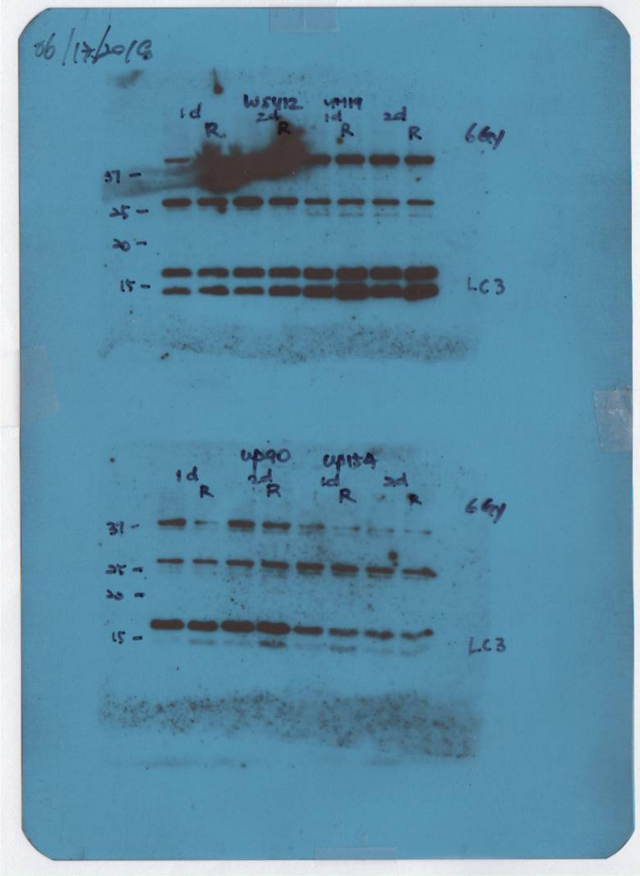

HPV(-)

LC3

HPV(+)

LC3

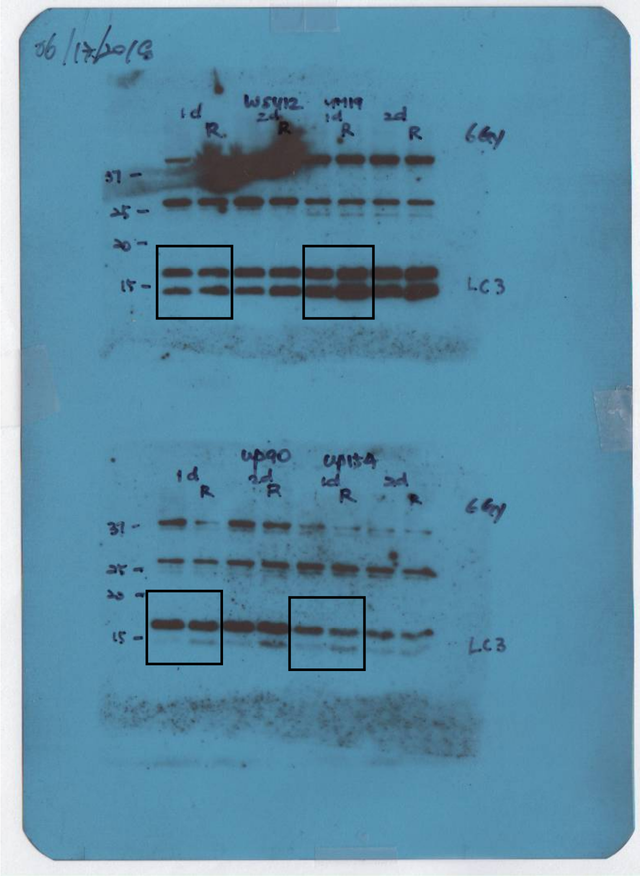

Figure 1C

p62

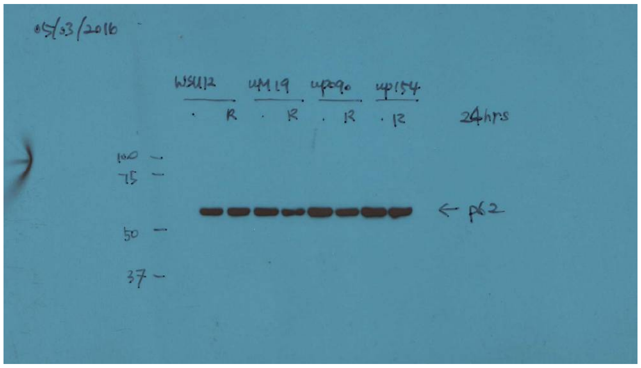

p62

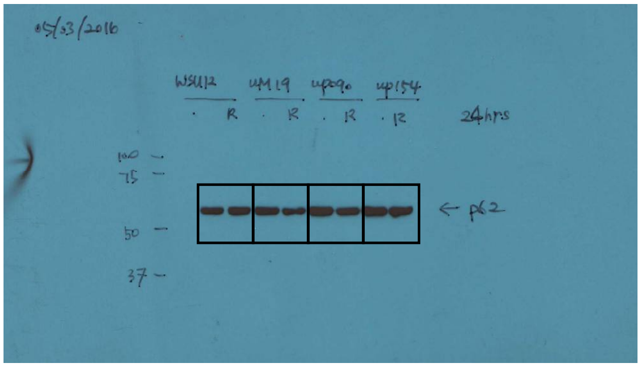

GAPDH

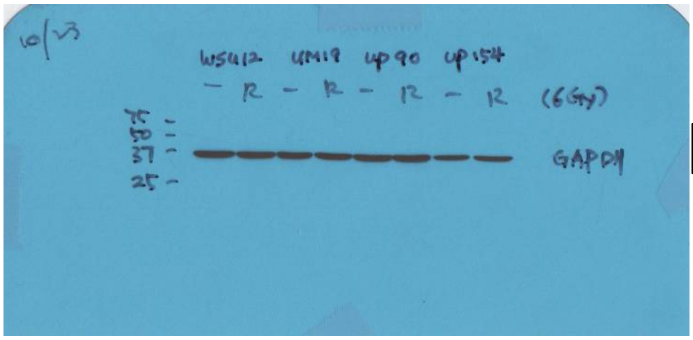

GAPDH

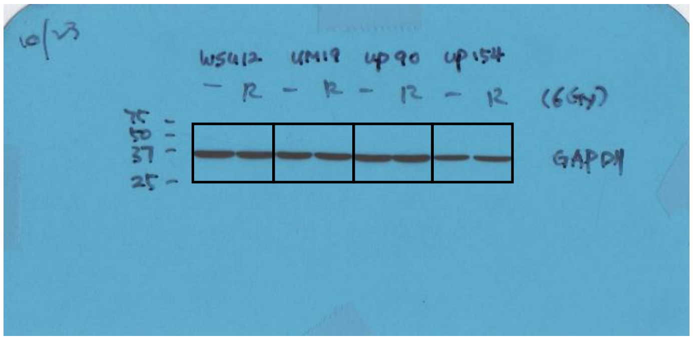

Figure 3A

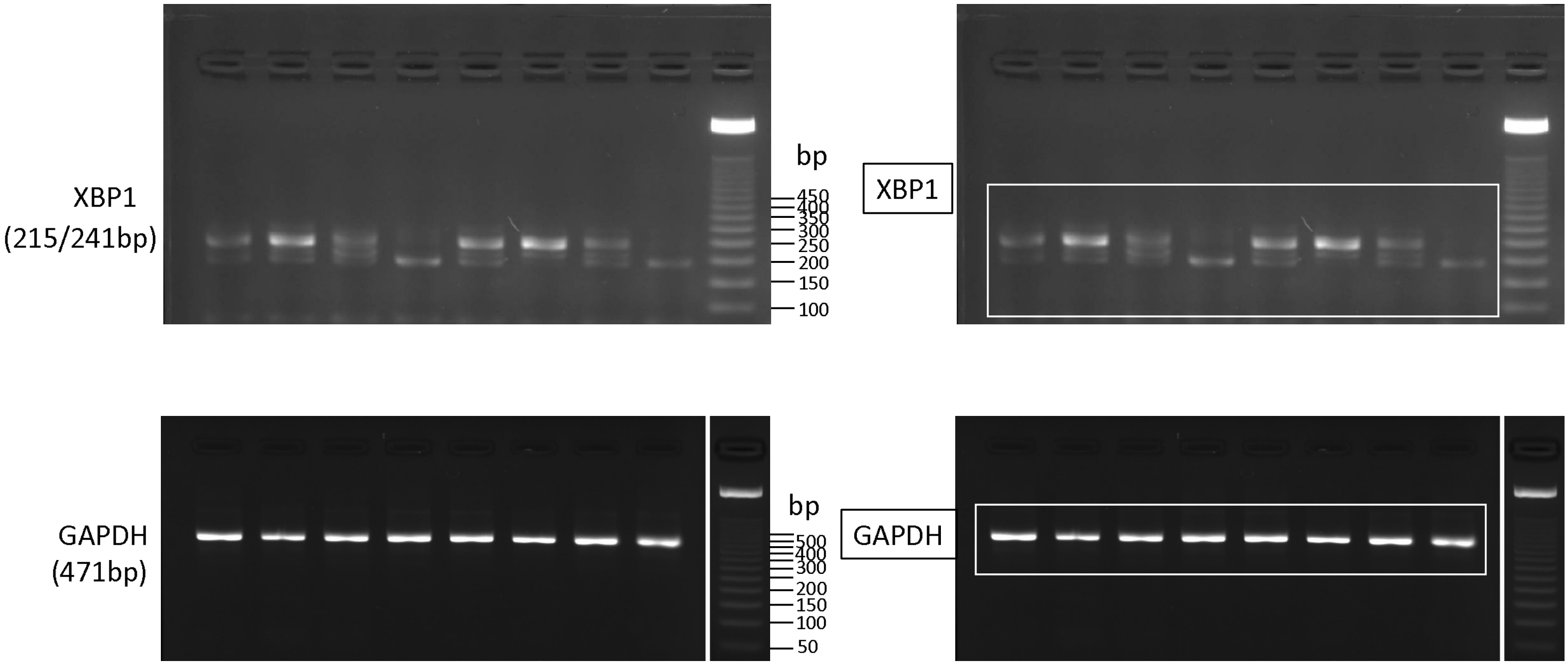

350

250

Figure 3B

Bip

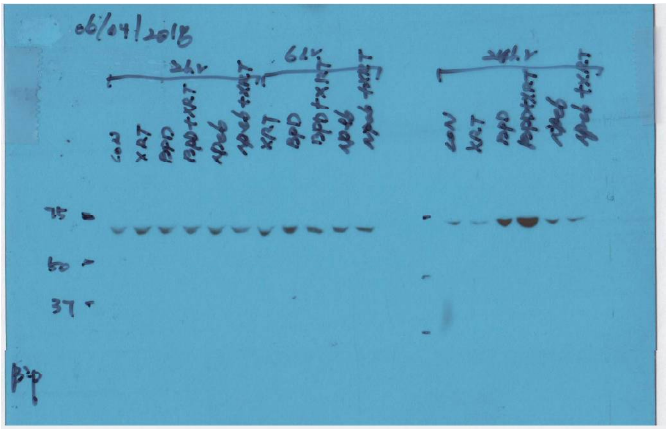

Bip

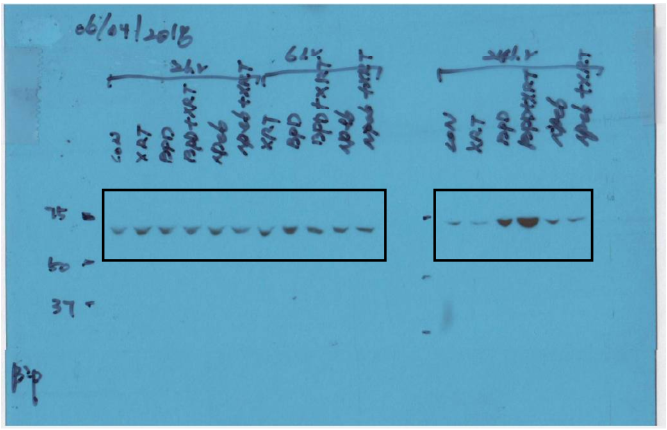

Figure 3B

CHOP

CHOP

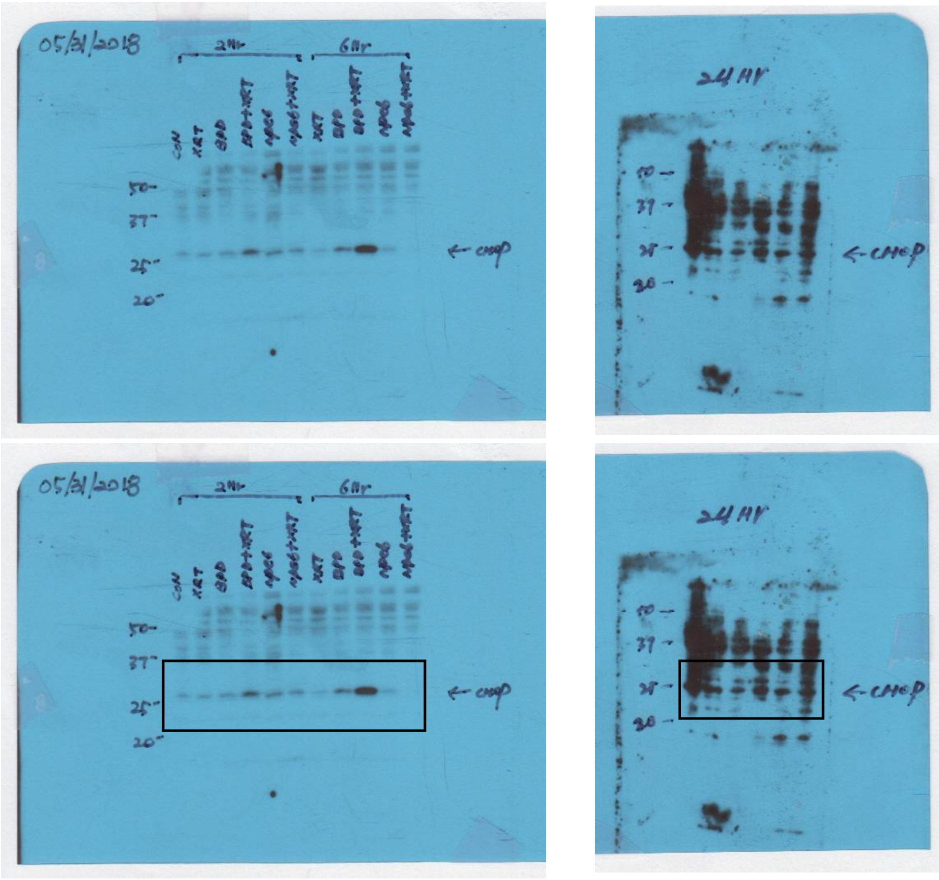

Figure 3B

Bcl-XL

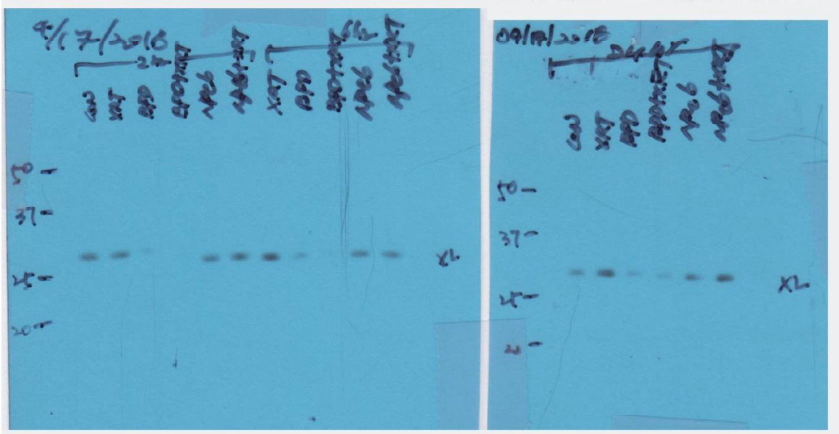

Bcl-XL

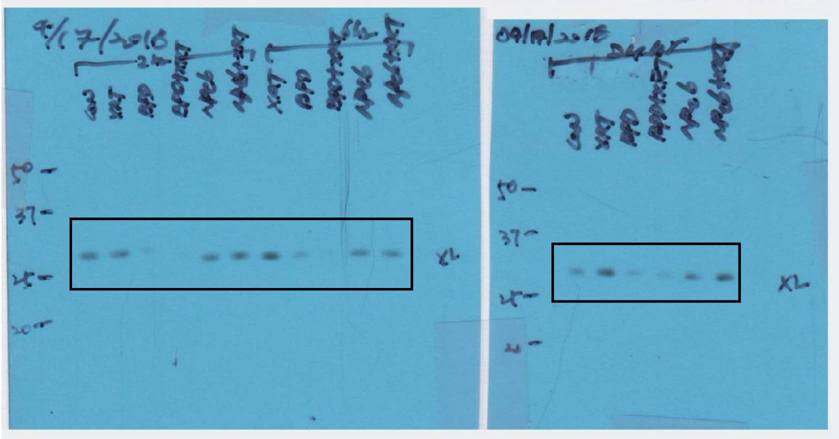

Figure 3B

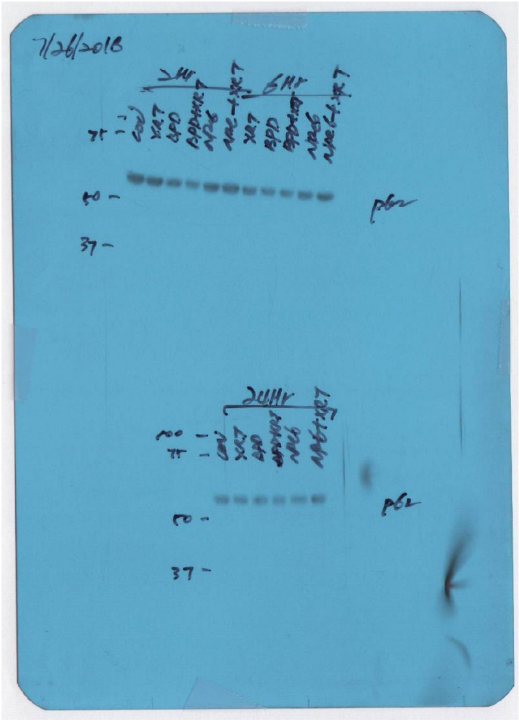

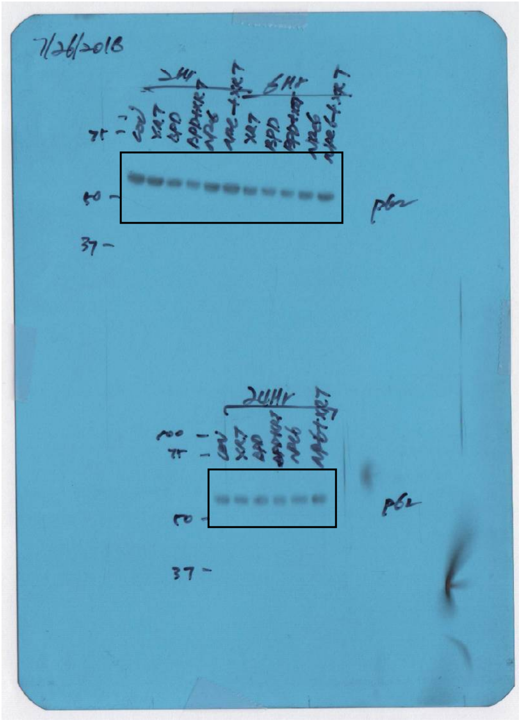

Figure 3B

LC3

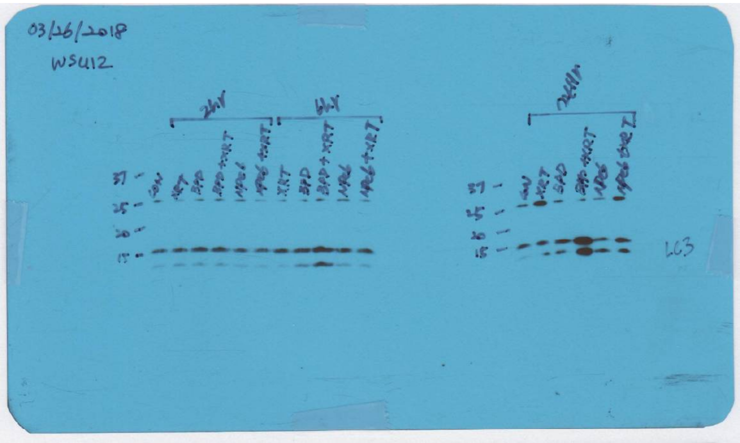

LC3

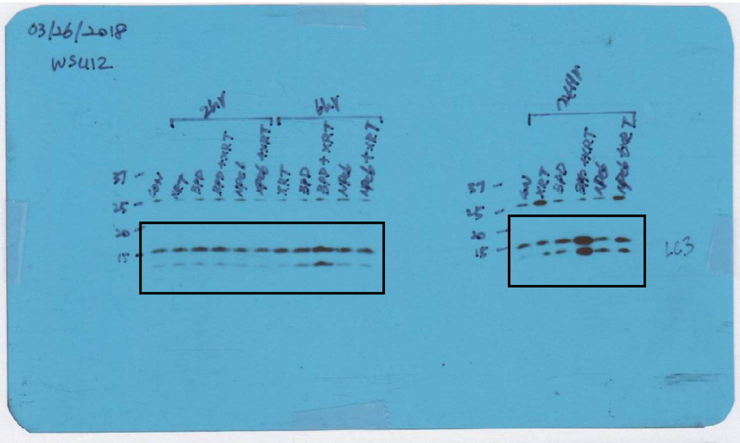

Figure 3B

GAPDH

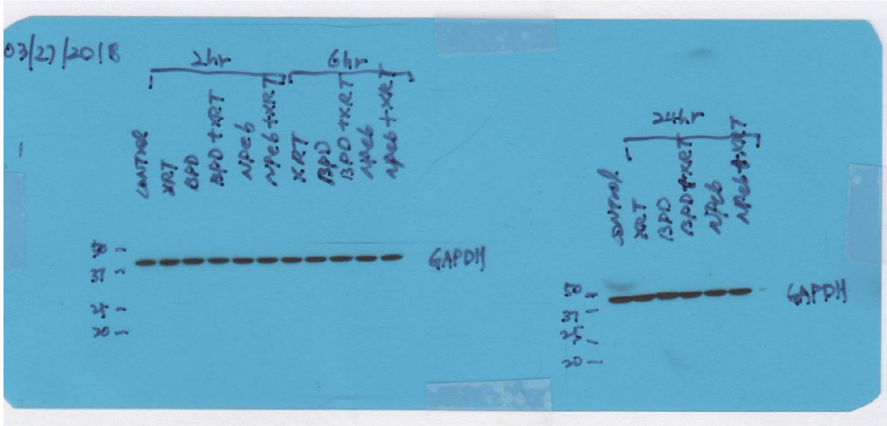

GAPDH

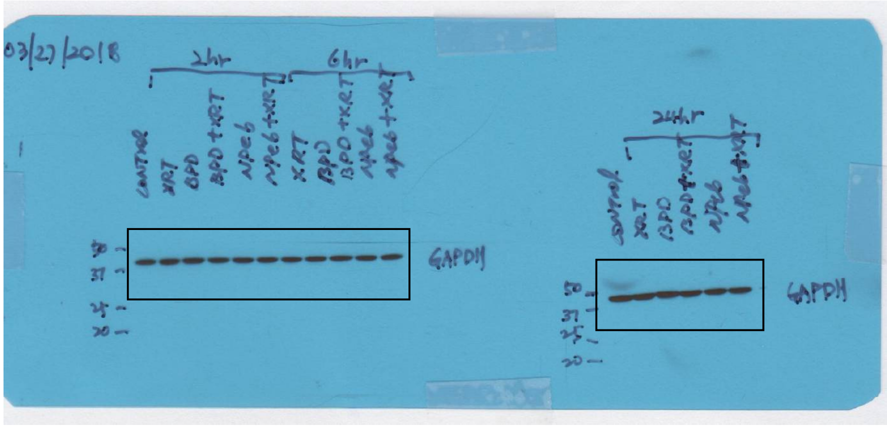

Figure 3C

p-JNK

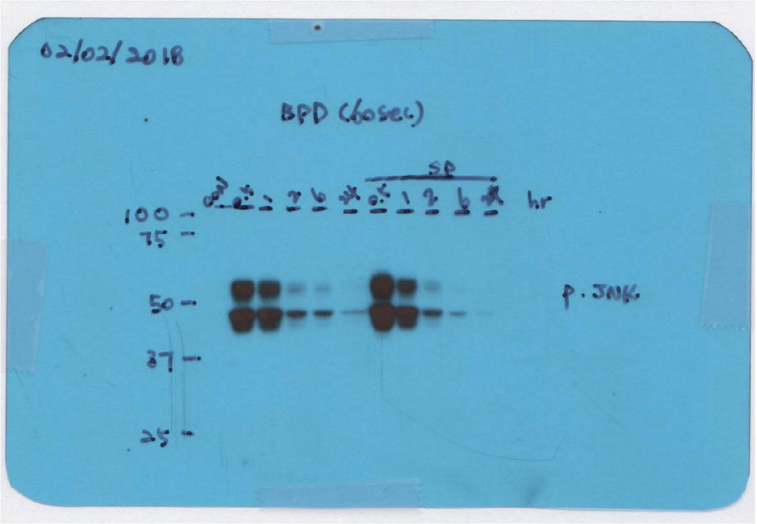

p-JNK BPD

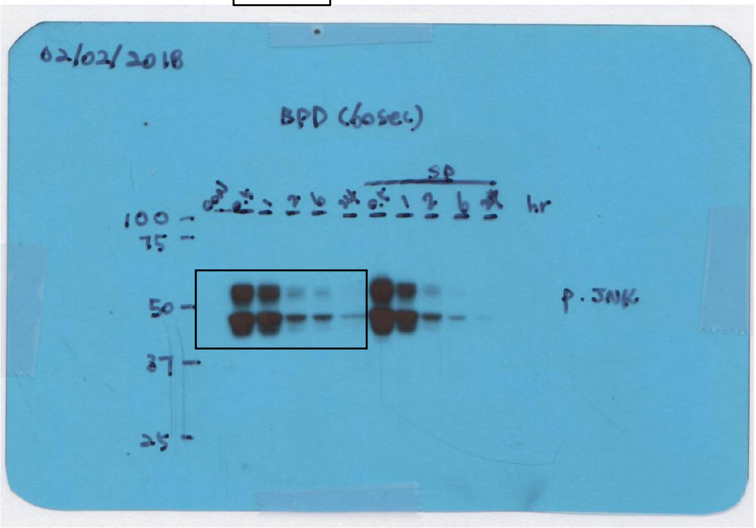

Figure 3C

t-JNK

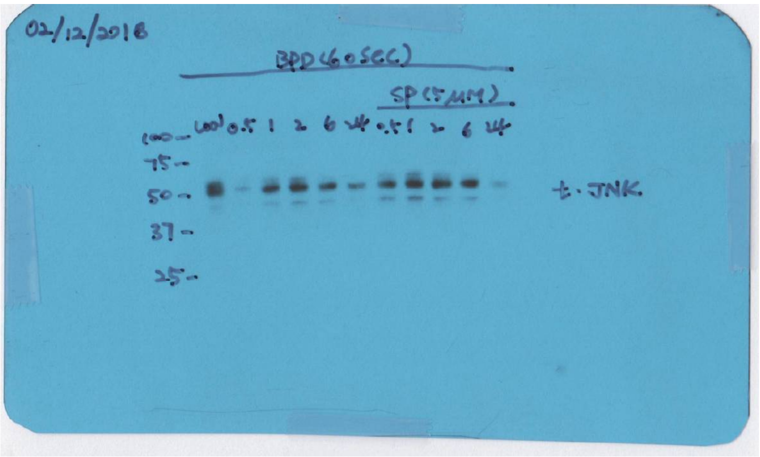

t-JNK BPD

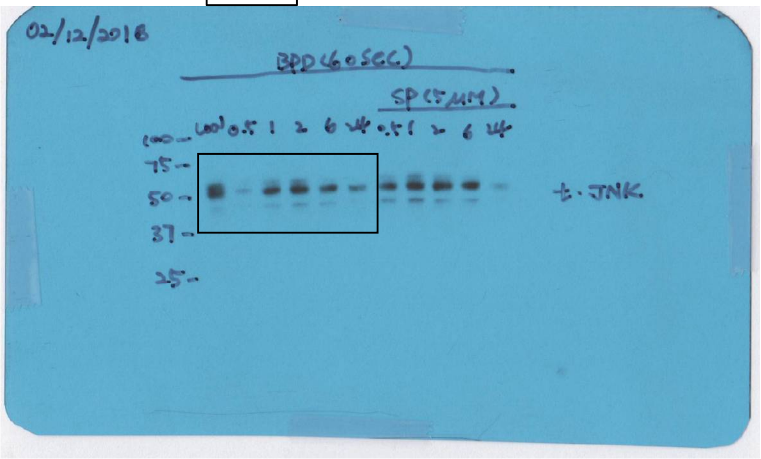

Figure 3C

p-p38

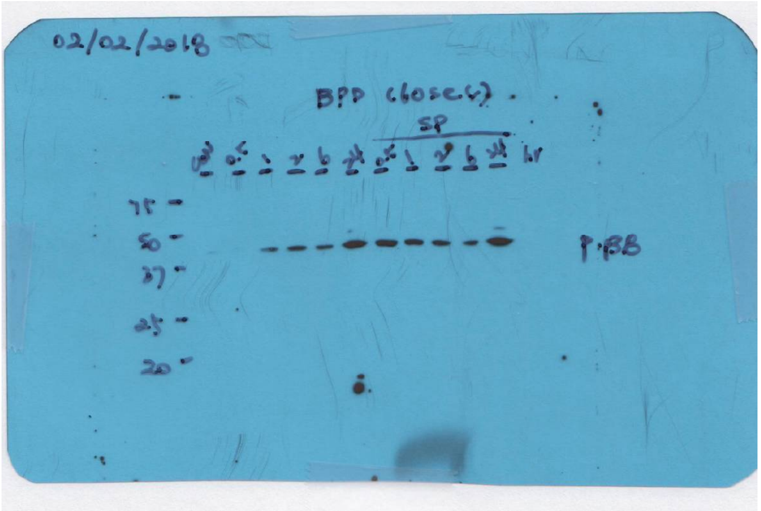

p-p38 BPD

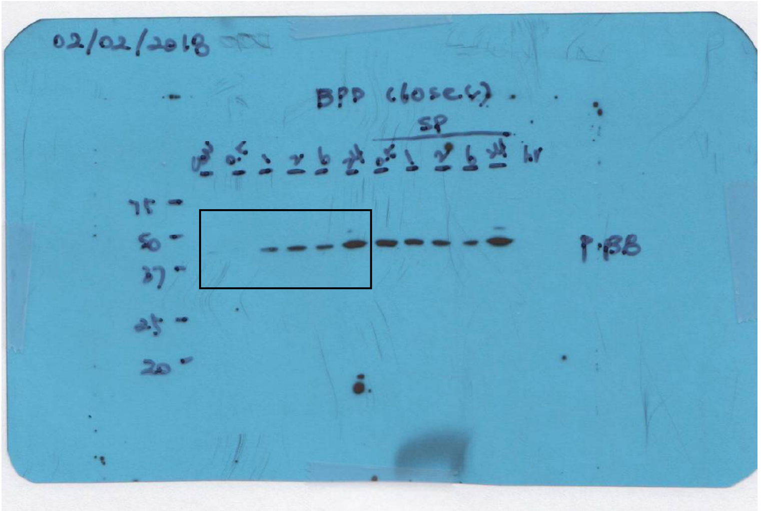

Figure 3C

t-p38

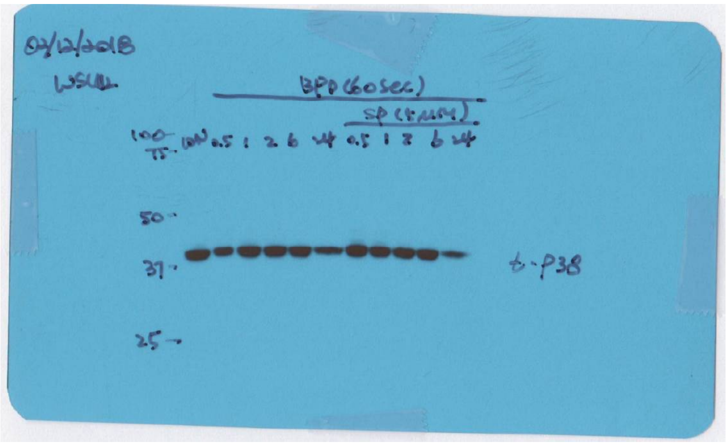

t-p38

BPD

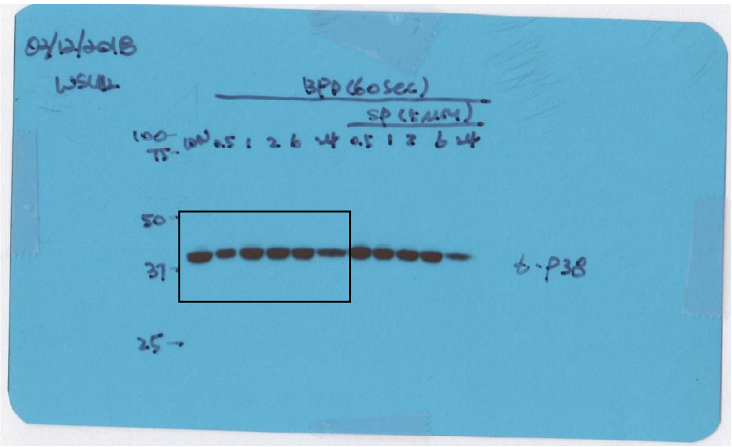

Figure 3C

GAPDH

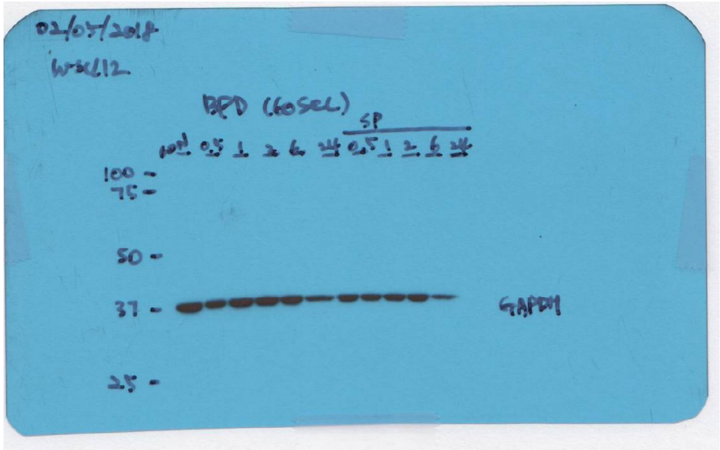

GAPDH BPD

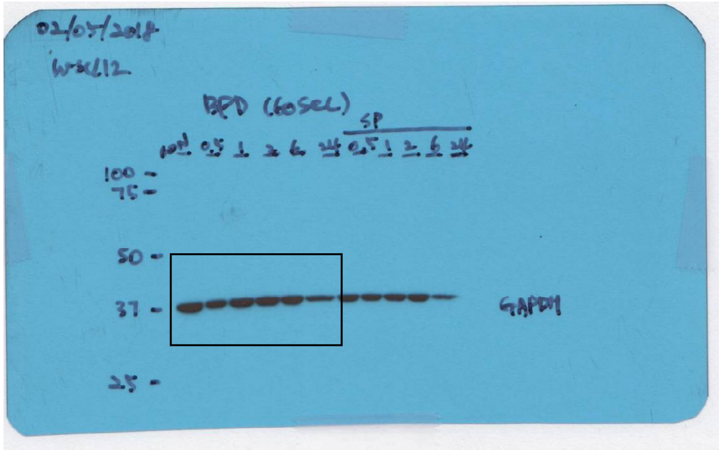

Figure 4A

LC3

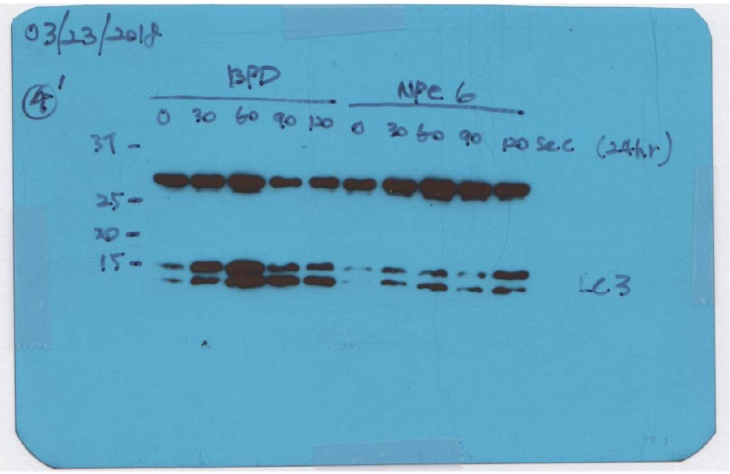

LC3 BPD

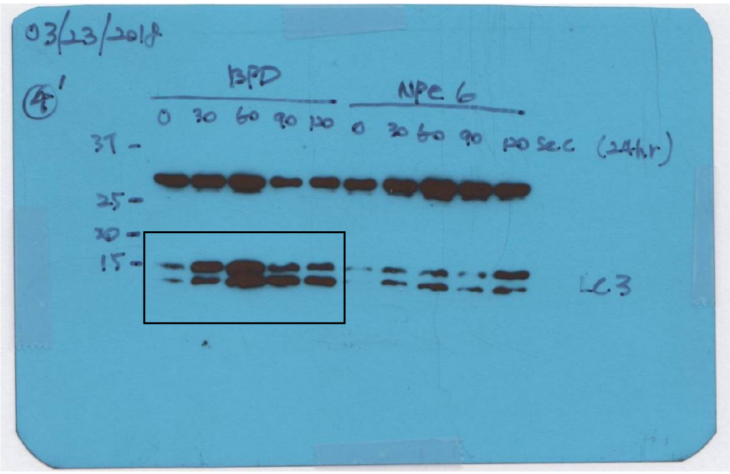

Figure 4A

GAPDH

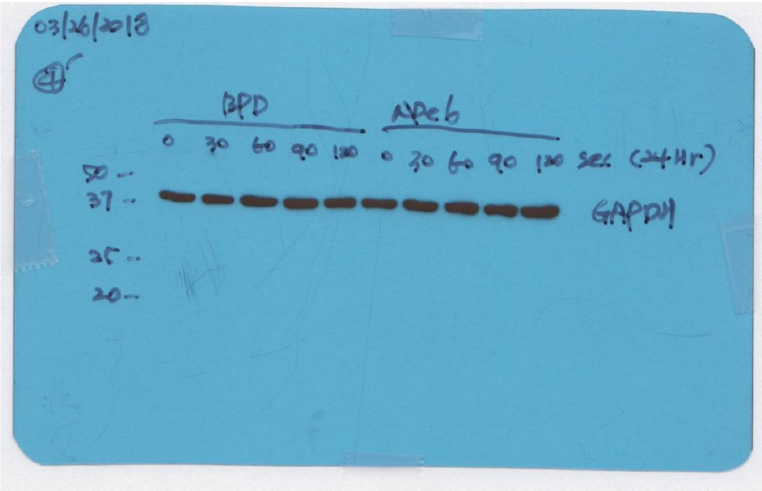

GAPDH BPD

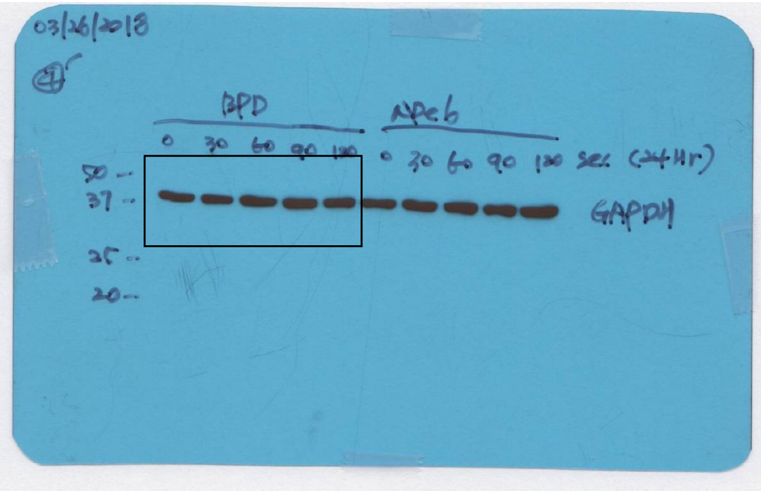

Figure 4A

LC3

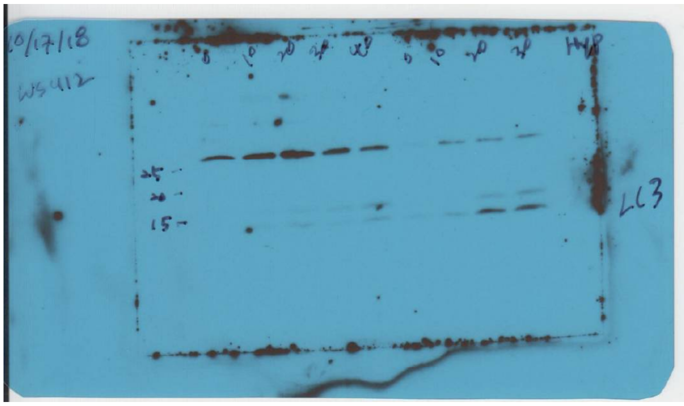

LC3

Hypericin

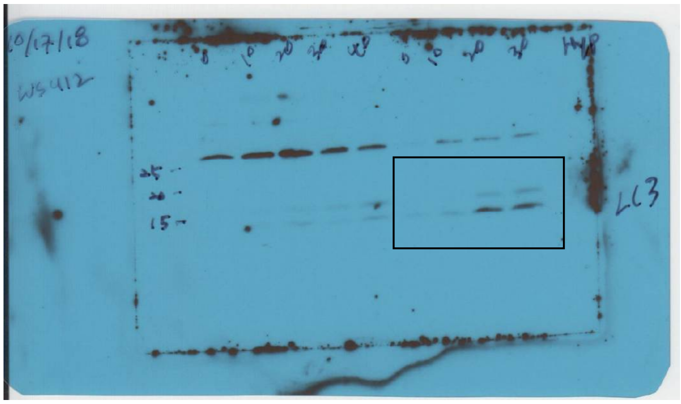

Figure 4A

GAPDH

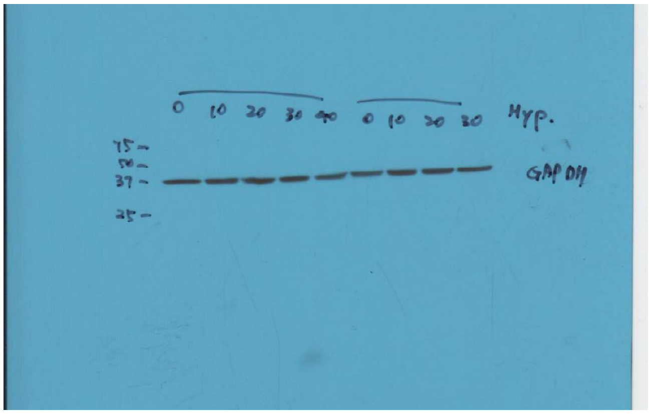

GAPDH Hypericin

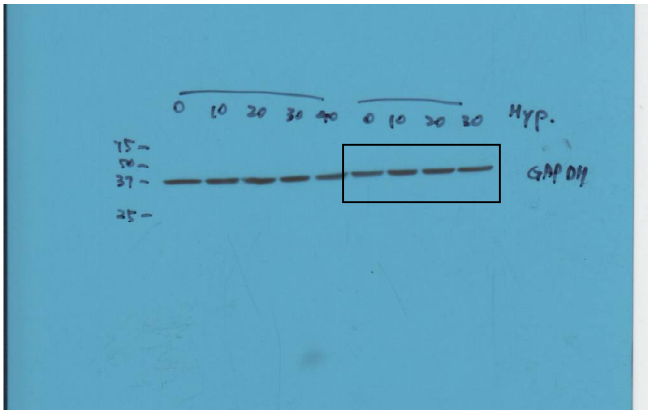

Figure 4 B

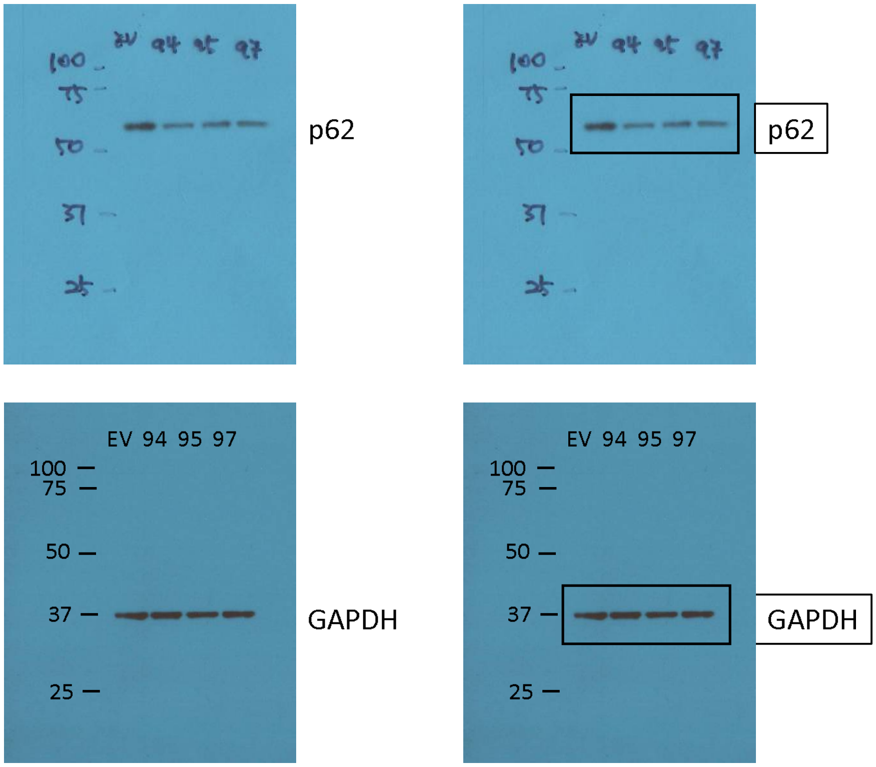

Supplement: Supplementary file 1 [file cancers-13-01193-s001.zip › cancers-1125978-Supplementary materials-figureS6.pdf]
